# *ABIOTIC STRESS GENE 1* mediates aroma volatiles accumulation by activating MdLOX1a in apple

**DOI:** 10.1093/hr/uhae215

**Published:** 2024-08-08

**Authors:** Jing Zhang, Yongxu Wang, Susu Zhang, Shuhui Zhang, Wenjun Liu, Nan Wang, Hongcheng Fang, Zongying Zhang, Xuesen Chen

**Affiliations:** College of Chemistry and Material Science, Shandong Agricultural University, Tai’an, 271018, Shandong, China; Shandong Institute of Pomology, Shandong Academy Agricultural Sciences, Tai’an, 271000, Shandong, China; College of Horticulture Sciences and Engineering, Shandong Agricultural University, Tai’an, 271018, Shandong, China; College of Horticulture Sciences and Engineering, Shandong Agricultural University, Tai’an, 271018, Shandong, China; College of Horticulture Sciences and Engineering, Shandong Agricultural University, Tai’an, 271018, Shandong, China; College of Horticulture Sciences and Engineering, Shandong Agricultural University, Tai’an, 271018, Shandong, China; College of Forestry, Shandong Agricultural University, Tai’an, 271018, Shandong, China; College of Horticulture Sciences and Engineering, Shandong Agricultural University, Tai’an, 271018, Shandong, China; College of Horticulture Sciences and Engineering, Shandong Agricultural University, Tai’an, 271018, Shandong, China

## Abstract

Fruit aroma is an important organoleptic quality, which influences consumer preference and market competitiveness. Aroma compound synthesis pathways in plants have been widely identified, among the lipoxygenase pathway is crucial for fatty acid catabolism to form esters in apple. However, the regulatory mechanism of this pathway remains elusive. In this study, linear regression analysis and transgene verification revealed that the lipoxygenase MdLOX1a is involved in ester biosynthesis. Yeast one-hybrid library screening indicated that a protein, MdASG1 (ABIOTIC STRESS GENE 1), was a positive regulator of *MdLOX1a* and ester production based on yeast one-hybrid and dual-luciferase assays, as well as correlation analysis among eight different apple cultivars. Overexpression of *MdASG1* in apple and tomato stimulated the lipoxygenase pathway and increased the fatty acid-derived volatile content, whereas the latter was decreased by *MdASG1* silencing and CRISPR/Cas9 knockout. Furthermore, *MdASG1* overexpression enhanced the salt-stress tolerance of tomato and apple ‘Orin’ calli accompanied by a higher content of fatty acid-derived volatiles compared to that of non-stressed transgenic tomato fruit, while *MdASG1*-Cas9 knockdown calli do not respond to salt stress and promote the biosynthesis of fatty acid-derived volatiles. Collectively, these findings indicate that MdASG1 activates *MdLOX1a* expression and participates in the lipoxygenase pathway, subsequently increasing the accumulation of aroma compounds, especially under moderate salt stress treatment. The results also provide insight into the theory for improving fruit aroma quality in adversity.

## Introduction

Volatile organic compounds in plants play important roles during biotic and abiotic stress, and act as signals to attract or repel pests, confer resistance to pathogens, and participate in seed dispersal [1]. Many volatiles are synthesized by plants at different developmental stages, particularly during fruit ripening. A great number of volatile esters are produced by strawberries (*Fragaria vesca*), bananas (*Musa sapientum*), apples (*Malus domestica*), and peaches (*Prunus persica*) [2–4]. Fruit quality mainly reflects fruit shape, size, color, aroma, acidity, sugar content, and nutritional content. Among these traits, aroma is a crucial factor in affecting the commercial value of fruit. However, breeders tend to focus on yield, disease resistance, and fruit color, while paying little attention to flavor, which weakens customer motivation to buy apple fruit [5]. Therefore, improving fruit flavor is desirable to meet consumer demand.

The aroma compound synthesis pathway has been extensively studied in plants, mainly including terpenoid pathways, amino acid metabolism pathway, and fatty acid metabolism pathway. In apples, the β-oxidase and lipoxygenase pathways are the two main enzyme systems involved in fatty acid catabolism to form esters [6]. Aldehydes, alcohols, esters, and other volatiles derived from fatty acids are principally produced by the lipoxygenase pathway. However, the lipoxygenase pathway in tomatoes (*Solanum lycopersicum*) produces a range of volatiles derived from fatty acids, principally C5 or C6 aldehydes and alcohols [7]. The main synthesis processes in the lipoxygenase pathway are as follows: lipoxygenases (LOX) catalyze polyunsaturated fatty acids, including linolenic and linoleic acid, to produce hydroperoxides [8]. Subsequently, hydroperoxides are converted to short-chain aldehydes and an oxo-acid by hydroperoxide lyase (HPL) [9, 10]. Alcohol dehydrogenase (ADH) further reduces the short-chain aldehydes to matching alcohols during fruit ripening [10, 11]. Finally, to create esters, acyl-coenzyme A, an alcohol acceptor, and an acid donor are catalyzed by alcohol acyl-transferases (AATs) [12]. Lipoxygenases are non-heme iron-containing dioxygenases, classified as either 9-LOX or 13-LOX based on the carbon position for oxygenation in polyunsaturated fatty acid. Lipoxygenases are also categorized as type 1 or type 2 LOXs based on sequence similarity. Type 1 LOXs share about 70% sequence similarity, and type 2 LOXs share about 40% sequence similarity, which have a putative chloroplast transit peptide [13]. Lipoxygenases in tomato, pepino (*Solanum muricatum*), and kiwifruit (*Actinidia deliciosa*) are involved in the produce of aroma substances [14–17].

Certain other factors affect the accumulation of fruit flavor compounds, including genetic differences [18], crop management [19], harvest date [20], storage environment [21], and the plant hormones ethylene, jasmonic acid, and abscisic acid, [22–24]. Recently, the transcriptional regulation in aroma synthesis has been investigated. The EIL and NAC activate the terpene synthase gene *AaTPS1* transcription to control monoterpene production in kiwifruit (*Actinidia arguta*) [25]. NAC transcription factors modulate ester biosynthesis by controlling the expression of the structural genes *FAD1* and *AAT10* in kiwifruit [26, 27], and *AAT* expression in tomato, peach, and apple [4]. The AP2/ERF transcription factors EREB58, CitAP2.10, and CitERF71 may transactivate the terpene synthase *TPS* to promote the synthesis of terpenes [28–30]. Strawberry ethylene response factors FaERF9 and FaMYB98 form a protein complex, which indirectly activates strawberry quinone oxidoreductase (FaQR) expression, and promote furanone biosynthesis [31]. The MYB transcription factors FaEOBII and FaDOF2 synergistically regulate the volatile phenylpropanoid pathway in strawberries [32, 33]. In tomatoes, the RIN and SlMYB75 directly bind to the genes related to the aroma compound synthesis pathway to activate their expression [34, 35]. In addition, other transcription factors, such as MYC2, PAP1, and bZIP regulate aroma compound biosynthesis [36–38].

*ABIOTIC STRESS GENE 1* (*ASG1*) is an abiotic stress gene identified in *Solanum tuberosum* and *Arabidopsis thaliana,* which is induced by stress through an ABA-dependent pathway [39]. However, little information is available on whether *ASG1* mediates other biological activities, such as aroma regulation. Stress can induce the production of secondary metabolites to improve fruit quality. Treatment with ABA reduces tannin content and positively affects grape (*Vitis vinifera*) fruit quality [40]. Abscisic acid drives the accumulation of secondary metabolites that contribute to fruit aroma in grapes and strawberries [41, 42]. MdAREB2 is responsive to ABA and promotes the accumulation of soluble sugars by activating the expression of amylase and sugar transporter genes [43]. Soil water stress can improve fruit quality by increasing the soluble sugar content in kiwifruit and apple fruit [44, 45]. Drought treatment induces the accumulation of flavonoids and anthocyanins in apples [46]. Recently, transcriptome analysis of apricot fruit revealed that MYC and bHLH transcription factors may respond to stress and play a crucial role in flavor formation [47]. However, the regulatory mechanism of stress-mediated aroma accumulation remains unclear.

Apple (*M. domestica*) are widely grown and considered essential commercial trees [48, 49]. Roughly 350 volatile chemicals, including alcohols, aldehydes, terpenes, ketones, and esters, are produced by apple fruit as it ripens [50, 51]. About 20 types of volatile chemicals are characteristic of the apple aroma, including *trans*-2-hexenal, butyl acetate, hexanol, 2-methyl butyl acetate, and hexyl acetate [52]. With the ripening of fruit, the abundance of esters increases significantly [6, 53]. In the ‘Golden Delicious’ apple, 80% of the volatile fragrance components are made up of esters [54], and 23 functional LOXs have been identified, of which MdLOX1a and MdLOX5e may participate in volatile component biosynthesis [55]. In the lipoxygenase pathway, LOX genes are crucial. On the other hand, not much is known about how LOXs are regulated in apples.

In this study, we chose a ripening-related gene, *MdLOX1a*, to explore ester biosynthesis. This selection was based on the results of a correlation analysis and the overexpression of *MdLOX1a* in apple calli. An abiotic stress gene, *MdASG1*, was identified through yeast one-hybrid library screening. *MdASG1* directly bound to *MdLOX1a*, activating its transcript, and subsequently enhanced the synthesis of aroma compounds. Overexpression of *MdASG1* in tomato fruit increased the production of volatile aroma compounds under salt stress. Overall, the findings significantly advance our understanding of the regulatory mechanism of aroma biosynthesis in apple fruit and the improvement of fruit aroma quality through stress mediation.

## Results

### *MdLOX1a* is involved in ester formation in apples and phylogenetic analysis of LOXs

**Figure 1:**
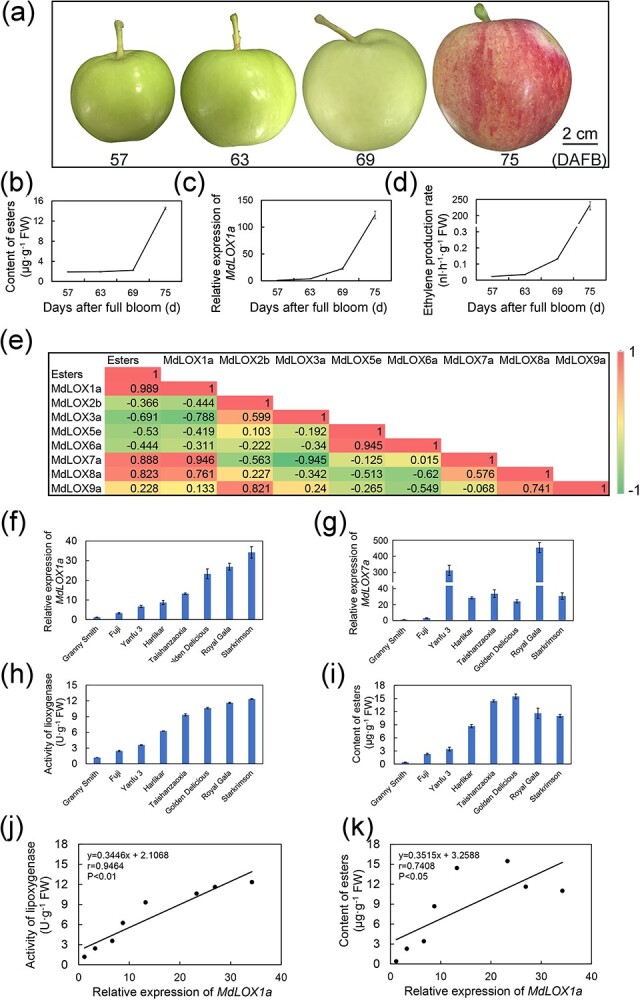
*MdLOX1a* is involved in ester formation in apples. **(a)** Apple ‘Taishanzaoxia’ fruit were harvested at 57, 63, 69, and 75 days after full bloom (DAFB). Bar = 2 cm. **(b)**–**(d)** Ester content **(b)**, transcript level of *MdLOX1a*  **(c)**, and ethylene release rate **(d)** during apple developmental stages. *MdActin* served as a control gene. Error bars represent the SD of three biological replicates. **(e)** Correlation analysis of *MdLOX* expression and ester content in apple fruit during the ripening stage. **(f)**–**(i)** Relative expression of *MdLOX1a*  **(f)** and *MdLOX7a*  **(g)**, lipoxygenase activity **(h)**, and ester content **(i)** in fruit of eight apple cultivars at the ripening stage. *MdActin* served as a control gene. Error bars represent the SD of three biological replicates. FW, Fresh weight. **(j)** Linear regression analysis between *MdLOX1a* expression and lipoxygenase activity in the fruit of eight apple cultivars. FW, Fresh weight. Significant differences were determined using Tukey one-way analysis of variance (ANOVA) with SPSS Statistics 22. **(k)** Linear regression analysis between *MdLOX1a* expression and ester content in the fruit of eight apple cultivars. FW, Fresh weight. Significant differences were determined using Tukey one-way analysis of variance (ANOVA) with SPSS Statistics 22.

We sampled apple fruit at four different developmental stages, including ripening stages (Fig. 1a) for gas chromatography–mass spectrometry (GC–MS) analysis. As the ripening process progressed, large amounts of esters were produced. In ripe fruit, the ester content reached about 14 μg·g^−1^ fresh weight, which was nearly seven times that of immature fruit at 57 days after full bloom (DAFB) (Fig. 1b; Table S1, see online supplementary material). In apples, eight groups of LOXs are employed in the lipoxygenase pathway to synthesize esters. To clarify the curial lipoxygenase genes employed in the ripening of fruit, we analysed eight lipoxygenase genes from each group using reverse transcription quantitative PCR (RT-qPCR) during fruit developmental stages (Fig. 1c; Fig. S1, see online supplementary material). As the fruit matured, the expression levels of *MdLOX1a* and *MdLOX7a* increased significantly, consistent with the ethylene release. Specifically, *MdLOX1a* transcript abundance increased about 122-fold at the ripening stage compared to that of immature fruit (57 DAFB) (Fig. 1c and d). The ester content and the *MdLOX1a* expression profile showed a strong positive connection throughout apple fruit developmental stages (*r* = 0.989, *P* < 0.05). Similarly, *MdLOX7a* also exhibited comparable results (*r* = 0.888, *P* < 0.05) (Fig. 1e; Fig. S2, see online supplementary material), suggesting that *MdLOX1a* and *MdLOX7a* may be maturity-related genes. To further analyse the relationship between *MdLOX1a/7a* and ester synthesis, we quantified *MdLOX1a* transcript levels (Fig. 1f), *MdLOX7a* transcript levels (Fig. 1g), lipoxygenase activity (Fig. 1h), and ester content (Fig. 1i) in ripe fruit of eight popular apple cultivars (Fig. S3, see online supplementary material). A positive connection was found between *MdLOX1a* expression and lipoxygenase activity (*r* = 0.9464, *P* < 0.01; Fig. 1j). Meanwhile, the *MdLOX1a* expression were positively correlated with ester content (*r* = 0.7408, *P* < 0.05) (Fig. 1k). However, the *MdLOX7a* transcript levels were not highly correlated with lipoxygenase activity (*r* = 0.4855) (Fig. S4, see online supplementary material) and ester content (*r* = 0.3422) (Fig. S5, see online supplementary material) in eight apple cultivars. These results illustrate that *MdLOX1a* may be an important gene involved in volatile ester biosynthesis.

Functional LOXs have been identified in various plant species, such as common bean (*Phaseolus vulgaris*), tomato, kiwifruit, Arabidopsis, grape, rice (*Oryza sativa*), persimmon (*Diospyros kaki*), and oriental melon (*Cucumis melo*) [14, 15, 56–61]. Furthermore, 23 functional LOXs in the lipoxygenase pathway have been identified in the genome of the ‘Golden Delicious’ apple [55]. In the present study, the amino acid sequences of 58 LOXs from 14 plant species were analysed. The LOXs were mainly divided into 9-LOXs and 13-LOXs, respectively. *MdLOX1a* and *MdLXO7a*, which exhibited similar expression patterns during apple fruit development and ripening, were clustered with 9-LOXs (Fig. S6, see online supplementary material). Furthermore, the sequence similarity between MdLOX1a and MdLOX7a proteins was 66.59% (Fig. S7, see online supplementary material), and they were also categorized as type 1 LOXs.

### Function analysis of MdLOX1a in aroma biosynthesis and its subcellular localization

**Figure 2:**
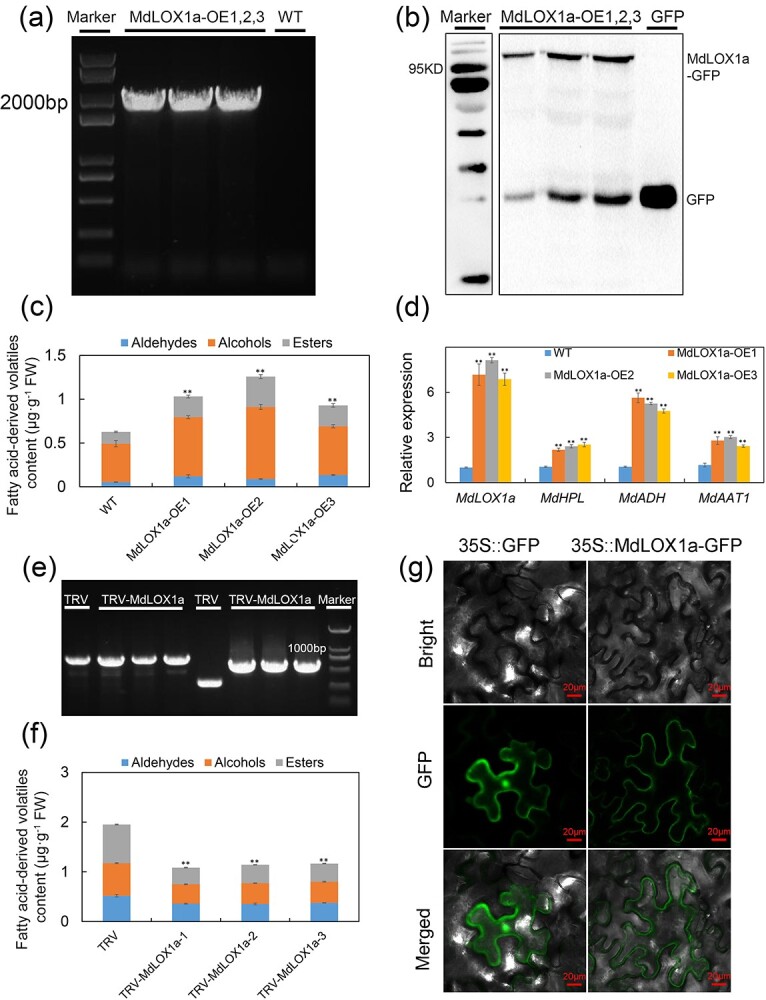
The role of *MdLOX1a* in apple fatty acid-derived volatile biosynthesis and subcellular localization of MdLOX1a. **(a)** and **(b)**  *MdLOX1a* overexpression in ‘Orin’ calli was verified by PCR amplification **(a)** and western blotting **(b)**. The 35S and MdLOX1a-PRI101-R primers were used to verify the transformants. **(c)** Fatty acid-derived volatile compounds in WT and *MdLOX1a*-overexpressing apple calli (MdLOX1a-OE). Error bars represent the SD of three biological replicates. FW, Fresh weight. Significant differences were detected by a two-sided Student’s *t*-test. (^*^P < 0.05 and ^**^P < 0.01). **(d)** Relative expression of fatty acid-derived volatile biosynthesis genes in WT and MdLOX1a-OE transgenic apple calli. *MdActin* served as a control gene. Error bars represent the SD of three biological replicates. Significant differences were detected by a two-sided Student’s *t*-test. (^*^P < 0.05 and ^**^P < 0.01). **(e)** Transient silencing of *MdLOX1a* was confirmed by PCR amplification. The TRV1-F and TRV1-R primers were used in lanes 1–4 from the left, and the TRV2-F and TRV2-R primers were used in lanes 5–8 from the left. **(f)** Fatty acid-derived volatile content in apple fruit with transient silencing of *MdLOX1a* (TRV-MdLOX1a) and the vector (TRV). Error bars represent the SD of three biological replicates. Significant differences were detected by a two-sided Student’s *t*-test (^*^P < 0.05 and ^**^P < 0.01). **(g)** Subcellular localization of MdLOX1a. MdLOX1a was mainly expressed in the cytoplasm of tobacco leaves. Bars = 20 μm.

To analyse the function of *MdLOX1a* in volatile aroma biosynthesis, we generated *MdLOX1a*-overexpressing transgenic ‘Orin’ calli (Fig. 2a and b). The levels of fatty acid-derived volatiles, such as 1-penten-3-ol, 1-hexanol, 2-ethyl-1-hexanol, ethyl 2-methylbutanoate, and 2-octanol acetate, were significantly higher compared to those of the WT (Fig. 2c; Table S2, see online supplementary material). In addition, the synthetic genes corresponding to the lipoxygenase pathway were up-regulated (Fig. 2d, see online supplementary material). In addition, we transiently silenced *MdLOX1a* (Fig. 2e). In fruits silenced with *MdLOX1a*, the opposite results were observed, as the levels of fatty acid-derived volatile contents were significantly inhibited at the TRV-MdLOX1a injection sites (Fig. 2f). Taken together, these findings show that *MdLOX1a* is associated with ester content. The construct 35S::MdLOX1a-GFP was generated to determine the subcellular localization of MdLOX1a. Strong green fluorescence signals were detected in the cytoplasm of tobacco (*Nicotiana benthamiana*) leaves (Fig. 2g), consistent with the subcellular localization predicted using Cell-PLoc 2.0 (Fig. S8, see online supplementary material).

### MdASG1 is a direct regulator of *MdLOX1a* and activates its expression

**Figure 3:**
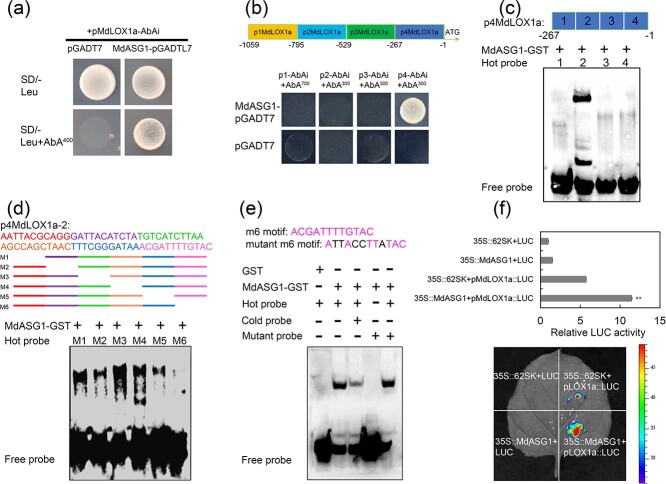
MdASG1 binds to *MdLOX1a* and activates its expression. **(a)** Yeast one-hybrid assays demonstrate binding between MdASG1 and *MdLOX1a* promoter. **(b)** Yeast one-hybrid assays demonstrate binding between MdASG1 and the fourth segment of the promoter of *MdLOX1a* (*p4MdLOX1a*). **(c)** Four segments of *p4MdLOX1a*. EMSA demonstrate binding of MdASG1 to the −201 ~ −135 bp segment of *p4MdLOX1a* (*p4MdLOX1a-2*). **(d)** Design of biotin-labeled probes (M1–M6) for the partial deletion of the fragment *p4MdLOX1a-2*. The M6 fragment showed no binding with MdASG1. **(e)** EMSA demonstrates MdASG1 binding to the m6 motif in *MdLOX1a*. Symbols + and − indicate the presence or absence of specific probes. The hot probe consisted of a biotin-labeled fragment. The cold probe consisted of an unlabeled fragment. The mutant probe contained five nucleotide mutations. **(f)** Dual-luciferase assay verify that MdASG1 transformation activated the *MdLOX1a* promoter. Error bars represent the SD of three biological replicates. Significant differences were detected by a two-sided Student’s *t*-test. (^*^P < 0.05 and ^**^P < 0.01).

Given that *MdLOX1a* is a crucial gene in volatile ester biosynthesis, we used the *MdLOX1a* promoter as bait to conduct yeast one-hybrid library screening. We identified a protein, designated MdASG1 (accession number: XM_029093686), that can bind to the promoter of *MdLOX1a* under 400 ng·mL^−1^ aureobasidin A (AbA) (Fig. 3a, Fig. S9, see online supplementary material). The amino acid sequence of MdASG1 showed 70% and 73% similarity with Arabidopsis AtASG1 and potato (*Solanum commersonii*) ScASG1, respectively (Fig. S10, see online supplementary material). Both *AtASG1* and *ScASG1* respond to stress treatment [39]. The promoter of *MdLOX1a* was divided into four fragments in order to identify the specific binding site of MdASG1. MdASG1 bound to the *p4MdLOX1a* fragment in yeast one-hybrid assays (Fig. 3b). An electrophoretic mobility shift assay (EMSA) showed that only the region from –201 to −135 bp of *p4MdLOX1a* (*p4MdLOX1a-2*) contained a binding site (Fig. 3c). Partial deletion of the fragment *p4MdLOX1a-2* was performed to generate six individual fragments. Interestingly, binding was not observed in the absence of the m6 region (Fig. 3d). Therefore, we concluded that the specific binding motif of MdASG1 was located in the m6 region. The addition of a cold probe weakened the binding. When the binding sites were changed, the binding disappeared (Fig. 3e). The dual-luciferase reporter assay was conducted to elucidate that MdASG1 functions as a transcriptional activator targeting *MdLOX1a* (Fig. 3f).

### Correlation of *MdASG1* expression with *MdLOX1a* transcript level and ester content

**Figure 4:**
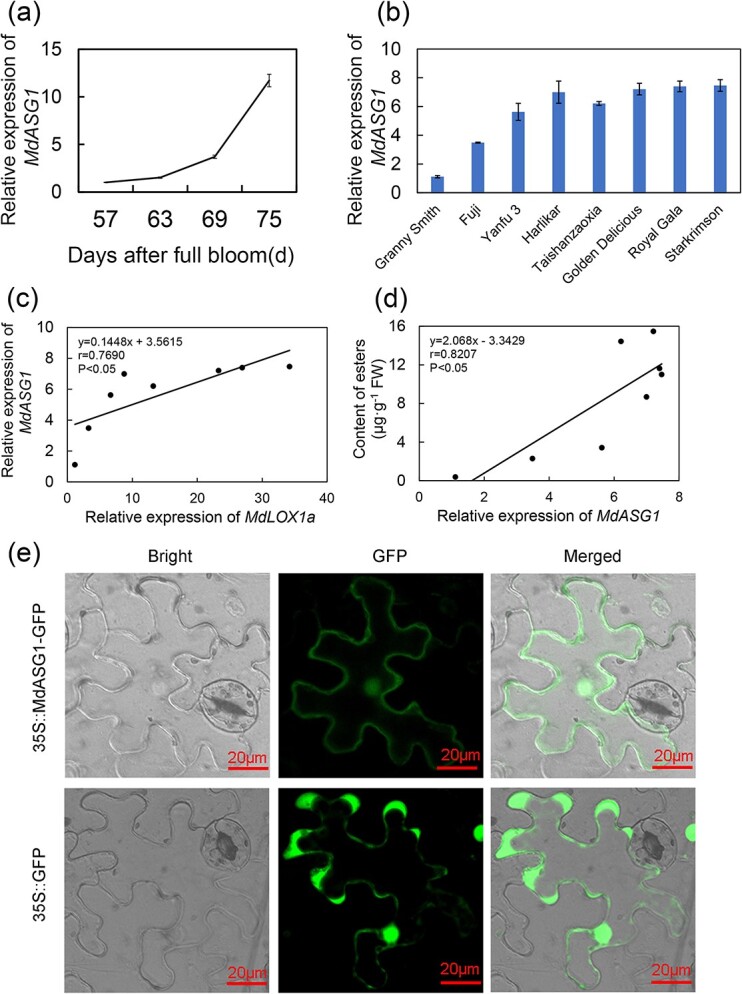
*MdASG1* is involved in ester biosynthesis in apple. **(a)** and **(b)** Transcript levels of *MdASG1* during apple developmental stages **(a)**, and in fruit of eight popular apple cultivars at ripening **(b)**. *MdActin* served as a control gene. Error bars represent the SD of three biological replicates. **(c)** Linear regression analysis between *MdLOX1a* expression and *MdASG1* expression in the fruit of eight different apple cultivars. Significant differences were determined using Tukey one-way analysis of variance (ANOVA) with SPSS Statistics 22. **(d)** Linear regression analysis between ester content and *MdASG1* expression in the fruit of eight different apple cultivars. Significant differences were determined using Tukey one-way analysis of variance (ANOVA) with SPSS Statistics 22. **(e)** Subcellular localization of MdASG1. Bars = 20 μm.

To discover more about the connection between *MdASG1* expression and aromatic compound synthesis, we analysed the expression of *MdASG1* during apple fruit developmental stages (Fig. 4a), which was consistent with the ester content. Subsequently, the expression profile of *MdASG1* among eight apple cultivars was examined (Fig. 4b). Correlation analysis among the cultivars revealed that *MdASG1* expression was positively connected with *MdLOX1a* expression (*r* = 0.7690, *P* < 0.05) (Fig. 4c). Furthermore, the expression profile of *MdASG1* was correlated with ester content among the cultivars (*r* = 0.8207, *P* < 0.05) (Fig. 4d). Taken together, these findings show that *MdASG1* is a candidate gene in the lipoxygenase pathway. Subcellular localization showed that MdASG1 was uniformly distributed in all subcellular compartments (Fig. 4e).

### Changes in fatty acid-derived volatile content caused by transient overexpression of *MdASG1* or silencing of *MdASG1* in apple

**Figure 5:**
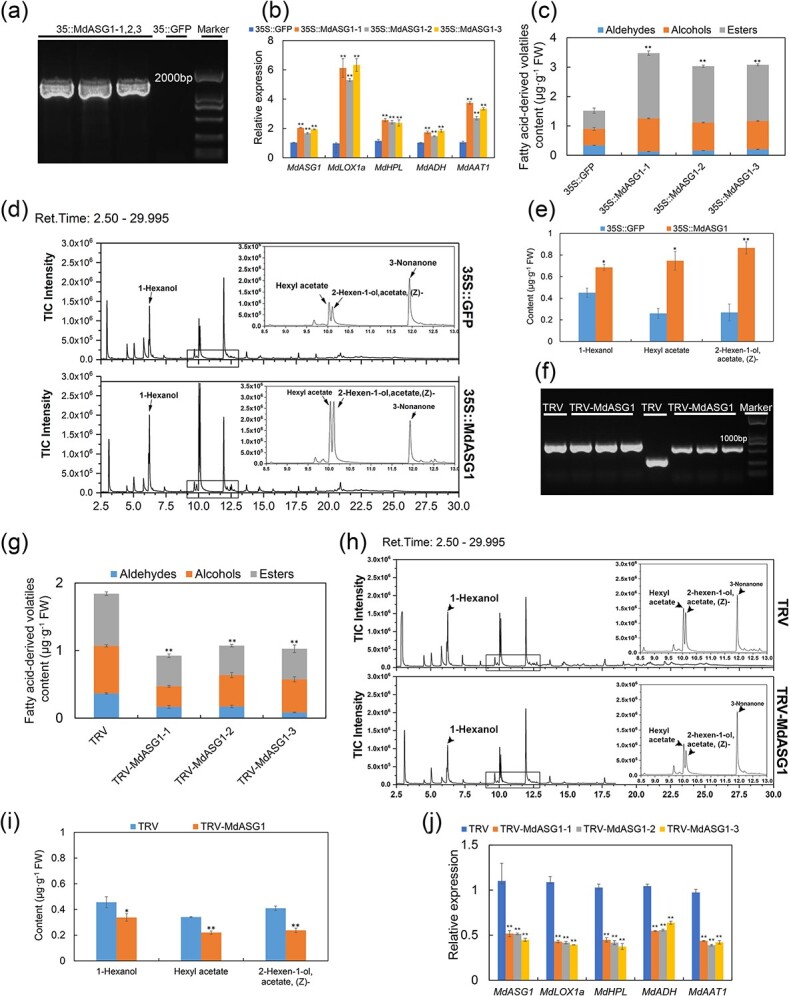
Transient overexpression or silencing of *MdASG1* in apple fruit. **(a)** Transient overexpression of *MdASG1* was confirmed by PCR amplification. The GFP-F and MdASG-PHB-R primers were used to verify the transformants. **(b)** Relative expression of *MdASG1* and genes related to fatty acid-derived volatile biosynthesis in apple with transient overexpression of *MdASG1* (35S::MdASG1) and the empty vector (35S::GFP). *MdActin* served as a control gene. Error bars represent the SD of three biological replicates. Significant differences were detected by a two-sided Student’s *t*-test (^*^P < 0.05 and ^**^P < 0.01). **(c)** Fatty acid-derived volatile content in 35S::GFP and 35S::MdASG1 transgenic apple fruit. Error bars represent the SD of three biological replicates. Significant differences were detected by a two-sided Student’s *t*-test (^*^P < 0.05 and ^**^P < 0.01). **(d)** Total Ion Chromatography (TIC) of 35S::GFP and 35S::MdASG1 transgenic apple fruit. **(e)** Contents of 1-hexanol, hexyl acetate, and 2-hexen-1-ol, acetate, (Z) in 35S::GFP and 35S::MdASG1 transgenic apple fruit. Error bars represent the SD of three biological replicates. Significant differences were detected by a two-sided Student’s *t*-test (^*^P < 0.05 and ^**^P < 0.01). **(f)** Transient silencing of *MdASG1* was confirmed by PCR amplification. The TRV1-F and TRV1-R primers were used in lanes 1–4 from the left, and the TRV2-F and TRV2-R primers were used in lanes 5–8 from the left. **(g)** Fatty acid-derived volatile content in apple fruit with transient silencing of *MdASG1* (TRV-MdASG1) and the vector (TRV). Error bars represent the SD of three biological replicates. Significant differences were detected by a two-sided Student’s *t*-test (^*^P < 0.05 and ^**^P < 0.01).**(h)** TIC of TRV and TRV-MdASG1 transgenic apple fruit. **(i)** Contents of 1-hexanol, hexyl acetate, and 2-hexen-1-ol, acetate, (Z) in TRV and TRV-MdASG1 transgenic apple fruit. Error bars represent the SD of three biological replicates. Significant differences were detected by a two-sided Student’s *t*-test. (^*^P < 0.05 and ^**^P < 0.01). **(j)** Relative expression of *MdASG1* and genes related to fatty acid-derived volatile biosynthesis in TRV and TRV-MdASG1 transgenic apple fruit. *MdActin* served as a control gene. Error bars represent the SD of three biological replicates. Significant differences were detected by a two-sided Student’s *t*-test. (^*^P < 0.05 and ^**^P < 0.01).

Given the positive correlation between the expression of *MdASG1* and *MdLOX1a*, as well as the ester content (Fig. 4), we hypothesize that *MdASG1* may regulate aroma compound biosynthesis. In order to test this hypothesis, we transiently overexpressed *MdASG1* in ‘Yinv’ apple by injecting *Agrobacterium tumefaciens* infiltration buffer containing the target gene or the empty vector (Fig. 5a). An approximately 2-fold increase in *MdASG1* transcript levels was observed, followed by about a 6-fold increase in *MdLOX1a* transcript levels (Fig. 5b). These changes were accompanied by higher contents of fatty acid-derived volatiles, including 1-hexanol, hexyl acetate, and 2-hexen-1-ol, acetate, (Z), compared with transient expression of the empty vector 35S::GFP (Fig. 5c–e). In addition, we transiently silenced *MdASG1* (Fig. 5f). The opposite results were observed in *MdASG1*-silenced fruits, where the levels of fatty acid-derived volatile contents were significantly inhibited at the TRV-MdASG1 injection sites (Fig. 5g). The main volatiles of apples, including 1-hexanol, hexyl acetate, and 2-hexen-1-ol, acetate, (Z) were significantly lower than those of the control (Fig. 5h and i). Silencing of *MdASG1* led to a corresponding decrease in the transcript levels of genes associated with the lipoxygenase pathway (Fig. 5j).

### Changes in fatty acid-derived volatile content caused by stable overexpression of *MdASG1*

**Figure 6:**
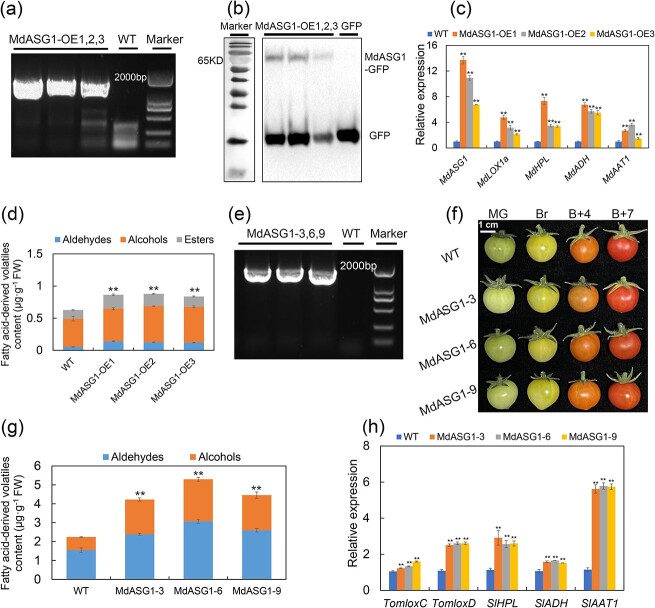
Stable overexpression of *MdASG1* in apple and tomato fruit. **(a)** and **(b)**  *MdASG1* overexpression in apple ‘Orin’ calli was verified by PCR amplification **(a)** and western blotting **(b)**. The 35S and MdASG1-PRI101-R primers were used to verify the transformants. **(c)** Relative expression of *MdASG1* and genes involved in fatty acid-derived volatile biosynthesis in *MdASG1*-overexpressing ‘Orin’ (MdASG1-OE) and WT calli. *MdActin* served as a control gene. Error bars represent the SD of three biological replicates. Significant differences were detected by a two-sided Student’s *t*-test (^*^P < 0.05 and ^**^P < 0.01). **(d)** Fatty acid-derived volatile compounds in WT and *MdASG1*-overexpressing apple calli (MdASG1-OE). Error bars represent the SD of three biological replicates. FW, Fresh weight. Significant differences were detected by a two-sided Student’s *t*-test (^*^P < 0.05 and ^**^P < 0.01). **(e)**  *MdASG1* overexpression in tomato verified by PCR amplification. The 188F and MdASG1-PCB302-R primers were used to verify the transformants. **(f)** Fruit of tomato ‘Micro-Tom’ overexpressing *MdASG1*. MG, mature green; Br, breaker; B + 4, 4 days after breaker stage; B + 7, 7 days after breaker stage. **(g)** Fatty acid-derived volatile content in the fruit of wild-type Micro-Tom (WT) and *MdASG1*-overexpressing tomato (MdASG1–3,6,9). Error bars represent the SD of three biological replicates. Significant differences were detected by a two-sided Student’s *t*-test (*P < 0.05 and ^**^P < 0.01). **(h)** Relative expression of fatty acid-derived volatile biosynthesis genes in fruit of WT and MdASG1–3,6,9 transgenic tomato plants. *SlActin* was used as an internal control gene. Error bars represent the SD of three biological replicates. Significant differences were detected by a two-sided Student’s *t*-test (^*^P < 0.05 and ^**^P < 0.01).

To provide additional evidence of *MdASG1*-mediated production of fatty acid-derived volatiles, we generated *MdASG1*-overexpressing ‘Orin’ calli (Fig. 6a and b). Overexpression of *MdASG1* leaded to upregulation of *MdLOX1a* expression level and that of other genes in the lipoxygenase pathway compared with the control calli (WT) (Fig. 6c). We further analysed *MdASG1*-overexpressing ‘Orin’ calli through GC–MS and found that the content of fatty acid-derived volatiles was significantly increased compared to that of the WT (Fig. 6d). To rapidly generate transgenic fruit, we overexpressed *MdASG1* in tomato ‘Micro-Tom’ and obtained the lines MdASG1–3, MdASG1–6, and MdASG1–9 (Fig. 6e and f). Ripening fruit of these overexpression lines exhibited elevated levels of volatiles compared to the wild type (Fig. 6g). The transcript levels of the synthase genes associated with the lipoxygenase pathway in transgenic tomato fruit were markedly higher than those of WT tomato (Fig. 6h). To summarize, these findings indicate that MdASG1 enhances the biosynthesis of fatty acid-derived volatiles by upregulating the transcript of *MdLOX1a* in the lipoxygenase pathway.

### Overexpression of *MdASG1* confers enhanced salt tolerance and accumulation of higher levels of fatty acid-derived volatiles under salt treatment

**Figure 7:**
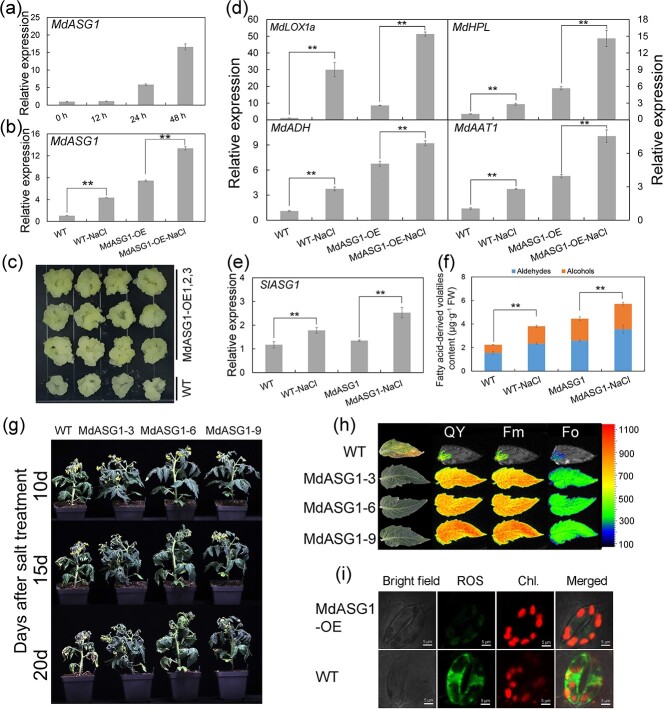
*MdASG1* enhances plant salt tolerance and mediates the accumulation of fatty acid-derived volatiles under salt stress. **(a)** Relative expression of *MdASG1* in wild-type tissue-cultured apple plantlets under 200 mM NaCl treatment. *MdActin* served as a control gene. Error bars indicate the SD of three biological replicates. **(b)** Transcriptional changes of *MdASG1* in WT and *MdASG1*-overexpressing transgenic lines (MdASG1-OE) in response to 50 mM NaCl treatment. *MdActin* served as a control gene. Error bars represent the SD of three biological replicates. Significant differences were detected by a two-sided Student’s *t*-test (^*^P < 0.05 and ^**^P < 0.01). **(c)** WT and MdASG1-OE transgenic ‘Orin’ calli treated with 50 mM NaCl. **(d)** Transcriptional changes in genes involved in fatty acid-derived volatile biosynthesis under 50 mM NaCl treatment in WT and MdASG1-OE transgenic ‘Orin’ calli. MdActin served as a control gene. Error bars represent the SD of three biological replicates. Significant differences were detected by a two-sided Student’s t-test (^*^P < 0.05 and ^**^P < 0.01). **(e)** Transcriptional changes in SlASG1 under 200 mM NaCl treatment in ripening fruit of WT and MdASG1-overexpressing (MdASG1) tomato plants. SlActin served as a control gene. Error bars represent the SD of three biological replicates. Significant differences were detected by a two-sided Student’s t-test (^*^P < 0.05 and ^**^P < 0.01). **(f)** Changes in fatty acid-derived volatile content under 200 mM NaCl treatment in ripening fruit of WT and MdASG1 transgenic tomato. Error bars represent the SD of three biological replicates. Significant differences were detected by a two-sided Student’s t-test (^*^P < 0.05 and ^**^P < 0.01). **(g)** Phenotype of WT and MdASG1–3,6,9 transgenic tomato plants under 200 mM NaCl treatment for 10, 15, and 20 d. **(h)** Chlorophyll fluorescence in tomato leaves after NaCl treatment for 20 days. **(i)** Fluorescence of reactive oxygen species in tomato leaf cells after NaCl treatment for 20 d. Bars = 5 μm. Error bars represent the SD of three biological replicates. Asterisks indicate statistical significance (^*^P < 0.05 and ^**^P < 0.01).

**Figure 8:**
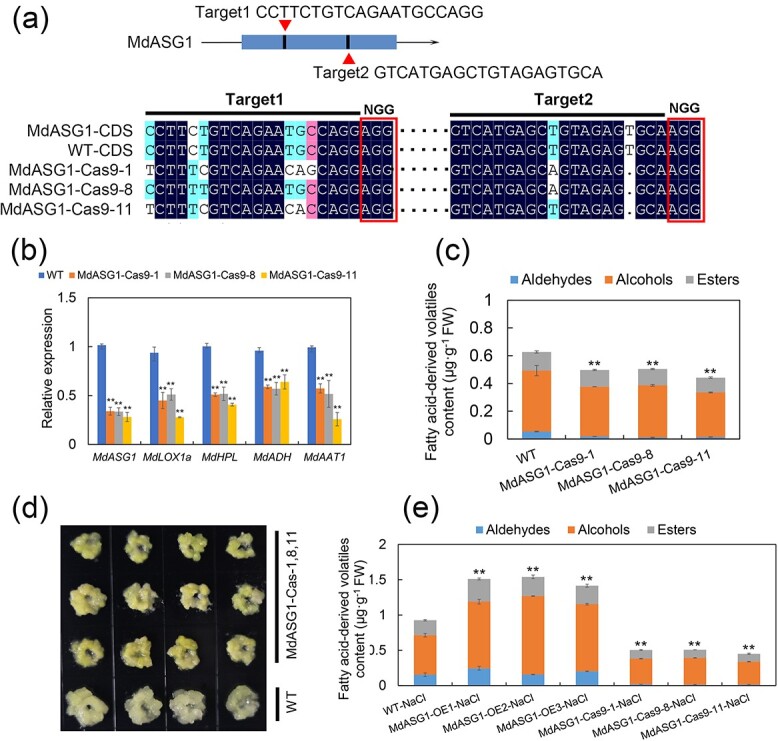
CRISPR/Cas9 knockdown *MdASG1* in ‘Orin’ calli and salt stress treatment. **(a)**  *MdASG1* knockdown sites design and sequencing results of *MdASG1*-Cas9 knockdown apple calli. Sequence alignment was performed using DNAMAN software, and the sequence before NGG was the knockout target. **(b)** Relative expression of *MdASG1* and genes involved in fatty acid-derived volatile biosynthesis in WT and *MdASG1*-Cas9 knockdown (MdASG1-Cas9) ‘Orin’ calli. *MdActin* served as a control gene. Error bars represent the SD of three biological replicates. Significant differences were detected by a two-sided Student’s *t*-test (^*^P < 0.05 and ^**^P < 0.01). **(c)** Fatty acid-derived volatile compounds in WT and MdASG1-Cas9 ‘Orin’ calli. Error bars represent the SD of three biological replicates. FW, Fresh weight. Significant differences were detected by a two-sided Student’s *t*-test (^*^P < 0.05 and ^**^P < 0.01). **(d)** WT and MdASG1-Cas9 transgenic ‘Orin’ calli were treated with 50 mM NaCl. **(e)** Changes in fatty acid-derived volatile content under 50 mM NaCl treatment in WT, MdASG1-OE and MdASG1-Cas9 transgenic ‘Orin’ calli. Error bars represent the SD of three biological replicates. FW, Fresh weight Significant differences were detected by a two-sided Student’s *t*-test (^*^P < 0.05 and ^**^P < 0.01).

*MdASG1* showed high homology with *AtASG1*. Therefore, we speculated that *MdASG1* may respond to abiotic stress similarly to *AtASG1*. As expected, *MdASG1* transcript levels were higher in response to NaCl treatment in tissue-cultured plantlets of ‘Royal Gala’ (Fig. 7a) and ‘Orin’ calli (Fig. 7b), particularly in *MdASG1*-overexpressing ‘Orin’ calli (Fig. 7b). Similarly, *MdASG1*-overexpression ‘Orin’ calli were more tolerant to salt stress than the control (Fig. 7c) and the transcription of stress-related genes was up-regulated (Fig. S11, see online supplementary material). Interestingly, the transcript levels of genes in the lipoxygenase pathway were up-regulated in response to 50 mM NaCl treatment for 20 days, particularly in calli overexpressing *MdASG1* (Fig. 7d). The levels of fatty acid-derived volatiles increased in response to the salt treatment in *MdASG1*-overexpressing ‘Orin’ calli (Fig. 8e). Similar results were found in tomatoes; the transcript levels of the tomato homolog *SlASG1* (Fig. 7e) and genes in the lipoxygenase pathway were up-regulated with 200 mM NaCl treatment in both transgenic tomato fruit and WT (Fig. S12, see online supplementary material). The levels of fatty acid-derived volatiles increased in response to the salt treatment in WT and transgenic tomato fruit (Fig. 7f). The transgenic tomato plants exhibited a significant increase in tolerance to salt stress (Fig. 7g), higher photosynthesis capacity (Fig. 7h), and reduced oxidative stress (Fig. 7i) compared with the WT. The expression of stress-related genes was upregulated in transgenic tomato plants (Fig. S13, see online supplementary material). In summary, these findings indicate that *ASG1* is involved in volatile compounds synthesis in apples and tomatoes, and higher levels of aroma compounds accumulate under salt stress.

### *MdASG1*-Cas9 knockdown calli do not respond to salt stress and promote the biosynthesis of fatty acid-derived volatiles

To further validate the involvement of MdASG1 in the synthesis of volatile aroma compounds in apples under salt stress, we generated MdASG1-knockdown ‘Orin’ calli (MdASG1-Cas9–1,8,11) using CRISPR-Cas9 technology. Through first-generation sequencing, we identified partial substitutions and deletions in the target sequences (Fig. 8a; Fig. S14, see online supplementary material), leading to a significant downregulation of *MdASG1* expression. Correspondingly, the expression levels of other genes in the lipoxygenase pathway and the content of fatty acid-derived volatiles were significantly downregulated compared with the control calli (WT) (Fig. 8b and c). Next, we performed salt stress on MdASG1-knockdown calli and found that *MdASG1*-Cas9 knockdown calli did not respond to salt stress (Fig. 8d). Meanwhile, the *MdASG1*-Cas9 knockdown calli accumulated lower levels of volatiles compared to the WT and MdASG1-OE transgenic ‘Orin’ calli under salt stress (Fig. 8e). In summary, it was shown that MdASG1 is involved in aroma chemicals synthesis in apples under salt stress.

## Discussion

Fruit flavor is the result of intricate interactions between aroma and taste [62]. Aroma is a blend of various volatile substances and is a crucial quality trait that influences consumer acceptance. During the ripening of apple fruit, the synthesis and accumulation of aroma compounds are increased, with esters constituting 80% of the volatiles [54, 63, 64]. The lipoxygenase pathway is responsible for esters synthesis, and the crucial enzyme involved is lipoxygenase. In pepino fruit during ripening, three LOX genes responsible for aroma compound biosynthesis, namely *SmLOXD*, *SmLOXB*, and *SmLOX5-like1*, are up-regulated [17]. In kiwifruit, *AdLox1* and *AdLox5* are up-regulated during ripening and are involved in fruity aroma esters biosynthesis [16]. *MdLOX1a* is associated with a quantitative trait locus for volatile esters in apples [65]. However, further study is needed to identify the function of *MdLOX1a* in ester synthesis. In the current study, *MdLOX1a* and *MdLOX7a* were up-regulated during fruit ripening, consistent with results reported by Schiller *et al.* [65]. We determined that *MdLOX1a* is involved in ester biosynthesis based on the significant positive connections between *MdLOX1a* expression and ester content. In addition, overexpression of *MdLOX1a* in apple ‘Orin’ calli led to an increase in the ester content. Therefore, we speculated that *MdLOX1a* is a crucial gene in the lipoxygenase pathway. Plant LOXs are localized in the cytoplasm or chloroplasts. TomloxC is involved in the synthesis of C5 and C6 flavor chemicals in tomato, which are localized in the chloroplasts [14, 66]. MdLOX1a is localized in the cytoplasm to participate in ester synthesis. Similarly, in kiwifruit, AdLox5 participates in the synthesis of fruity aroma esters in the cytoplasm [15, 16]. Phylogenetic analysis revealed that MdLOX1a can be classified as a 9-LOX. However, MdLOX1 is reported to have a dual positional specific function generating 9- and 13-hydroperoxides [65].

Transcriptional regulation of fruit aroma components has been widely reported in plants. However, previous research has mainly focused on terpene biosynthesis. For instance, several transcription factors of the MYC2, NAC, EIL, AP2/ERF, and MYB families [25, 28–30, 35, 36] are involved in terpene synthesis by directly activating the terpene synthase *TPS*. Recently, bZIP, NAC, and Dof families have been reported to play crucial roles in ester biosynthesis by regulating the expression of the structural gene *AAT* in the lipoxygenase pathway [4, 26, 27, 38]. *LOX* is a crucial structural gene in the lipoxygenase pathway, but the regulation of *LOX* is rarely reported. Given the observation that *MdLOX1a* mediates fruit ester biosynthesis, we used *MdLOX1a* as a candidate gene and identified an abiotic stress gene, *MdASG1*, which activates *MdLOX1a* expression by directly binding to its promoter. Furthermore, overexpression of *MdASG1* in fruit and calli increased the production of aroma compounds, while the synthesis of these compounds was reduced by silencing *MdASG1*. In *Saccharomyces cerevisiae*, the zinc cluster transcriptional regulator Asg1 is an activator of stress-responsive genes that are involved in fatty acid utilization [67]. However, MdASG1 and Asg1 of *S. cerevisiae* are entirely unrelated proteins.

The function of ASG (ScASG1 and AtASG1) was first identified in *S. tuberosum* and *A. thaliana*. It acts as a positive regulator of stress responses through an ABA-dependent pathway [39]. Amino acid sequence analysis revealed that MdASG1 showed high homology with Arabidopsis AtASG1 and potato ScASG1. In the current study, we observed a novel function for *ASG* in apples, mediating the biosynthesis of aroma compounds. In addition, we observed that *MdASG1* performed similar functions to those of *ScASG1* and *AtASG1* in response to NaCl treatment [39]. *MdASG1*-overexpressing calli and transgenic tomato plants (MdASG1–3,6,9) showed significantly improved tolerance to salt stress, higher photosynthesis capacity, and lower oxidative stress compared to the WT. However, *MdASG1*-knockdown calli were not tolerant to salt stress. We cloned *MdASG1* into the PHB vector and observed that MdASG1 was uniformly distributed in all subcellular compartments. In contrast, the potato ScASG1 is localized to the plasma membrane [39]. In Arabidopsis plants, overexpressing *DkLOX3* and *CaLOX1* increases tolerance to drought stress and severe salinity by modulating stress-related genes and reactive oxygen species production [60, 68]. In oriental melon, *CmLOX10* positively regulates drought tolerance [61]. In tomatoes, overexpression of ω-3 fatty acid desaturases (FAD) enhance tolerance to cold stress [69]. Therefore, we speculated that *MdASG1* might function by mediating the lipoxygenase pathway in response to abiotic stress.

Abiotic stress strongly affects plant growth. However, the observation that moderate stress may improve fruit quality is usually overlooked. Some previous studies have examined stress-mediated fruit quality, focusing mainly on sweetness and anthocyanin production, and less frequently on fruit aroma. For example, mild salt stress improves strawberry fruit quality by increasing the accumulation of sucrose and the antioxidant compounds anthocyanins and catechins [70–72]. Similarly, in tomatoes, NaCl treatment increases the concentration of soluble solids not only due to a reduction in water transport [73–75]. In grapes, moderate salinity increases anthocyanin and soluble solid contents but decreases aroma quality [76]. Conversely, in the present study, moderate salt stress increased the expression of lipoxygenase pathway-related genes in apple calli and tomato fruit, accompanied by an increased accumulation of aroma compounds. Especially in *MdASG1*-overexpressing apple calli and tomato, *MdASG1* further improved the content of fatty acid-derived volatiles under moderate salt stress. Banerjee *et al*. found that salt stress can promote aroma production in aromatic rice cultivars, except for Kalonunia [77]. At the same time, tomato *SlASG1*, which is a homolog of apple *MdASG1*, was significantly up-regulated under moderate salt stress, accompanied by an increase in aroma compound synthesis. When we knocked out *MdASG1* in calli, the *MdASG1*-knockdown calli did not respond to salt stress and did not promote the biosynthesis of fatty acid-derived volatiles. These results collectively indicate that *ASG1* is involved in salt-induced aroma biosynthesis through increased expression of genes in the lipoxygenase pathway.

The present results provide a theoretical foundation for the exploitation of moderate salt stress to improve fruit quality. This may facilitate the careful development and utilization of saline-alkali land to produce high-quality fruit. Rice and wheat (*Triticum aestivum* L.) are major crops grown worldwide, but their growth and yield are frequently constrained by salinity stress [78, 79]. According to estimates, salt stress affects at least 20% of all irrigated lands [80]. Considering that salinity may have a negative impact on irrigated areas, our findings may contribute to the improved utilization of saline-alkali land for fruit production.

## Conclusion

**Figure 9:**
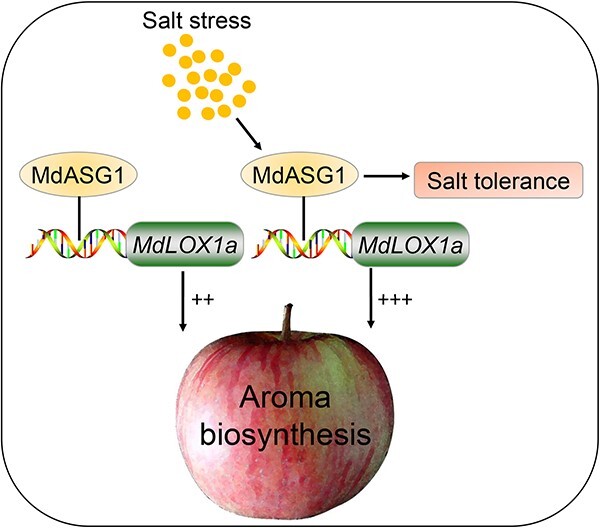
Proposed model for MdASG1 modulation of aroma compound biosynthesis in apples. MdASG1 can increase aroma compounds accumulation by activating *MdLOX1a* expression. Moreover, under moderate salt stress, MdASG1 enhances tolerance to salt stress and increases the produce of aroma compounds in fruit.

In summary, this study demonstrates the essential function of *MdLOX1a* in ester biosynthesis. We identified an abiotic stress gene, *MdASG1*, that directly binds to *MdLOX1a*, stimulating its transcription, and thereby contributing to the production of fatty acid-derived volatiles in apple fruit. Furthermore, *MdASG1* expression was upregulated under NaCl stress. *MdASG1*-overexpression calli and transgenic tomato plants (MdASG1–3,6,9) were more tolerant to salt stress than the WT. Transcript levels of genes in the lipoxygenase pathway were higher under salt stress compared to the non-stress conditions, which may elucidate how moderate stress improves fruit quality. The present results provide insight into the regulatory mechanism by which *MdASG1* directly triggers the expression of *MdLOX1a* to enhance the synthesis of aroma chemicals, particularly under moderate salt stress. (Fig. 9). Our findings establish a theoretical strategy for the production of high-quality apple fruit on moderately saline soil to meet consumer demands.

## Materials and methods

### Plant material

Apple ‘Taishanzaoxia’ fruits were picked at 57, 63, 69, and 75 DAFB from Liaocheng, Shandong Province, China. Fruit of eight apple cultivars was sampled at the ripening stage in Liaocheng, Shandong Province, China. The culture conditions of ‘Orin’ calli and ‘Royal Gala’ tissue-cultured plantlets were based on the description provided by Wang *et al*. [46]. Tomato ‘Micro-Tom’ plants were grown in a greenhouse at 24°C under a 16-hour light/8-hour dark photoperiod. The ‘Yinv’ fruit has been extensively used for transient transformation [81, 82]. In our study, we also utilized the ‘Yinv’ apple in the transient transformation assays. The fruits were harvested before ripening from trees in the germplasm nursery of the Shandong Institute of Pomology. *N. benthamiana* plant leaves (plant growth conditions: 25±1°C, 16-hour light/8-hour dark photoperiod) were employed for subcellular localization studies and dual-luciferase assays.

### Stress treatment

The shoot tip of 25-day-old ‘Royal Gala’ tissue-cultured plantlets was excised and transferred to liquid MS medium containing 200 mM NaCl. After 12, 24, and 48 h treatment, the shoots treated were immediately frozen in liquid nitrogen and stored at −70°C until needed. Transgenic (MdASG1-OE/Cas9) and control (WT) calli of uniform growth status were cultured on MS medium containing 50 μM NaCl for salt stress treatment for 20 days. Tomato plants grown in a square plastic pot (10 cm/ 7.5 cm / 8.5 cm at the top, the bottom, and height) were well-watered before the salt treatment. One-month-old tomato plants (WT and T_3_ transformants) of uniform growth were watered with a 200 mM NaCl solution at 4-day intervals until the fruits were ripe. After 20 days of salt treatment, the leaves were sampled to observe chlorophyll fluorescence, ROS, and for RNA extraction. The ripe tomato fruits were collected for GC–MS analysis. Three biological replicates were applied to all treatments mentioned above.

### Volatile collection and GC–MS analysis

The research method is based on Lu *et al*. [83], headspace solid-phase microextraction was utilized to analyse volatile chemicals in fruits. In brief, 5 g of fruit flesh tissue is diced, or 5 g of apple calli is ground into a paste, and transferred to a 50 mL conical flask. Tomato fruit required an additional 10 mL of saturated NaCl to extract volatile compounds. Subsequently, at the base of the conical flask, introduce 10 μL of 3-Nonanone standard solution (0.4 mg·mL^−1^, Sigma Aldrich, St. Louis, MO, USA) as the internal standard substance. The flask was sealed, and the extraction was carried out at 45°C for 40 minutes. The method for collecting and extracting volatile chemicals, along with the heating procedure of the GC–MS, were performed in accordance with the established procedure [83]. The NIST 2017 standard database was used to align the identified volatile chemicals. The volatile compound contents were quantified using the standard method outlined in the study. Three biological replicates were carried out for each experimental.

### RNA extraction and RT-qPCR

Total RNA was isolated from plant tissues using the FastPure® Plant Total RNA Isolation Kit (Vazyme, Nanjing, China). The cDNA synthesis was carried out using HiScript® II Reverse Transcriptase (Vazyme). RT-qPCR was conducted using the ChamQ SYBR qPCR Master Mix (Vazyme) on a CFX Connect instrument. The *MdActin* gene served as the internal control for apple samples, while the *SlActin* gene was utilized for tomato samples. Relative gene expression was quantified using the 2^−ΔΔ*C*t^ method [84]. Corresponding gene IDs are provided in Table S4 (see online supplementary material).

### Determination of lipoxygenase activity

Lipoxygenase activity was assessed utilizing a lipoxygenase assay kit (Mlbio, Shanghai, China). In brief, fruit tissue was pulverized into powder using liquid nitrogen. Subsequently, 0.1 g of the tissue was resuspended in 1 mL of extraction buffer, followed by centrifugation at 16000 × *g* for 20 minutes at 4°C. The resulting supernatant served as the enzyme extract. Enzyme activity was determined by combining 20 μL of enzyme extract, 160 μL of buffer solution reagent, and 20 μL of substrate solution. Lipoxygenase activity was quantified by measuring the increase in absorbance at 234 nm over a 1-minute period. One unit of enzyme activity was defined as a change in absorbance of 0.01 at 25°C per minute per gram of tissue. Each sample was analysed in triplicate to ensure reproducibility.

### Phylogenetic analysis

A phylogenetic tree was generated based on the multiple alignment of 58 LOX amino acid sequences derived from 14 plant species. The construction method is based on Zhang *et al.* [85]. The accession numbers for the LOX sequences are provided in Table S5 (see online supplementary material).

### Subcellular localization of MdLOX1a and MdASG1

The full-length CDS of *MdLOX1a* or *MdASG1* was cloned into the pHB vector, and subsequently transferred into GV3101 strain. The method of injection is based on Li *et al*. [86]. The fluorescence signal was imaged after infiltration for 2 days using a LSM800 confocal laser microscope (Carl Zeiss, Jena, Germany). The primers are provided in Table S6.

### Yeast one-hybrid assay

To screen for proteins that potentially bind to the promoter of *MdLOX1a*, we used the Matchmaker® Gold Yeast One-Hybrid Library Screening System (Clontech, Mountain View, CA, USA) following the manufacturer’s instructions. The *MdLOX1a* promoter (fragment length 1059 bp) was inserted into the pAbAi vector, and the linearized plasmid was transformed into the yeast strain Y1H Gold. The optimal AbA screening concentration was determined in accordance with the instructions. Total RNA extracted from ‘Taishanzaoxia’ apple fruit at various developmental stages was used to construct the prey cDNA library. The library plasmid (10 μL) was transformed into the MdLOX1a-pAbAi Y1H Gold strain to screen for the novel protein. In addition, the identified protein MdASG1 was inserted into the pGADT7 vector to confirm the result. The promoter of *MdLOX1a* was divided into four fragments (*p1MdLOX1a* to *p4MdLOX1a*) to identify the binding site. The primers are provided in Table S6.

### Dual-luciferase reporter assay

The full-length CDS of *MdASG1* was inserted into the pGreenII62-SK vector. The *MdLOX1a* promoter was inserted into the pGreenII0800-Luc vector. The assay method is based on Zhang *et al*. [87]. The In Vivo Imaging System (Xenogen, Alameda, CA, USA) was used to detect luminescence. The luciferase activities were measured using the Dual-Luciferase® Reporter Assay System (Promega, Madison, WI, USA).

### EMSA

The EMSA was conducted using the Lightshift Chemiluminescent EMSA kit (Thermo, New York, NY, USA). The full length CDS of *MdASG1* was inserted into the pGEX-4 T vector, and then introduced into BL21 strain to induce protein production. Subsequently, GST-tag Protein Purification Kit (Beyotime, Shanghai, China) was used to purify the protein. The biotin-labeled probe and MdASG1-GST protein were mixed in the binding buffer and incubated at 24°C for 15 minutes. As a control, GST protein was used, and unlabeled probes served as competitors. Table S7 lists the probes used in the EMSA assay.

### Fluorescence detection of reactive oxygen species

Reactive oxygen species were detected with fluorescent probes using a previously described method with slight modifications [46, 88]. Leaf discs were collected from transgenic and WT tomato plants after 20 days of salt treatment. The leaf discs were soaked in 0.01 mM PBS for 20 minutes, then placed in 10 μM 2′,7′-dichlorodihydrofluorescein diacetate (Invitrogen, Carlsbad, CA, USA) and incubated under vacuum for 30 minutes. Fluorescence signal was observed using a confocal laser microscope.

### Transient overexpression and gene silencing in apple fruit

Overexpression vector construction and infiltration of *MdASG1* were conducted as described for the subcellular localization assay. Virus-induced gene silencing was used to silence *MdASG1* and *MdLOX1a* in apple fruit. A partial CDS fragment for pTRV2-*MdASG1* (369 bp) and pTRV2-*MdLOX1a* (375 bp) were cloned by PCR using specific primers (Table S6). *A. tumefaciens* containing the target genes was injected into the epidermis of ‘Yinv’ apple fruit using a syringe. *A. tumefaciens* carrying the empty vector (PHB or TRV) served as the control group. Following infiltration, the fruits were incubated at 24°C with a 16-hour light/8-hour dark photoperiod in the incubator. Three days later, samples were collected from the fruit injection sites for transgene verification and RT-qPCR analysis. After 7 days, the fruit injection sites were sampled for volatile compound analysis using GC–MS. Three biological replicates with at least 15 fruits per group were analyzed.

### Stable overexpression in apple calli and tomato

The CDS of *MdLOX1a* and *MdASG1* were inserted into the PRI 101-AN vector. The recombinant plasmid was introduced into LBA4404 strain. Transformation of apple calli was conducted based on Zhang *et al*. [85]. The CDS of *MdASG1* was cloned into the PCB302 vector and then transferred into the LBA4404 strain. *Agrobacterium* infection solution with an OD_600_ of 0.6 was used to infiltrate tomato cotyledons for 15 minutes. To obtain resistant buds, the infiltrated cotyledons were screened on MS medium containing kanamycin (50 mg·L^−1^). Three lines were confirmed to be transgenic. Tomato fruits harvested at B + 7 days from WT and T_3_ transgenic plants were sampled for aroma compound analysis. Three biological replicates, each with 15 fruits, were analysed.

### Chlorophyll fluorescence analysis

Chlorophyll fluorescence parameters were measured using a Closed FluorCam FC800 chlorophyll fluorescence imaging system (Photon, Brno, Czech Republic). Before measurement, the leaves were dark-adapted for 30 minutes, then analysed to determine F_0_ (minimum fluorescence) and F_M_ (maximum fluorescence).

### CRISPR/Cas9 knockout of *MdASG1* in apple calli

The CRISPR/Cas9 knockout targets of MdASG1 were designed using the CRISPR-P 2.0 online tool. The knockout sequences were then ligated to the pHSE401 vector following the protocol by Zhang *et al*. [87]. Initially, single guide RNA (sgRNA) was obtained through cloning using primers listed in Table S6 (see online supplementary material) and the PCBC-DT1DT2 template. Next, the sgRNA mentioned above was attached to the pHSE401 vector. The recombinant plasmid was introduced into LBA4404 strain, and then transformed apple calli following the stable overexpression technique in apple calli. The transgenic calli were verified by PCR and sequencing. The expression levels of *MdASG1* were analysed using RT-qPCR with MdASG1-C primers (Table S3, see online supplementary material), which were designed within the knockout regions.

### Statistical analysis

The Student’s *t*-test was used to determine the significance of differences between two samples in this study. Figures were generated using Microsoft Excel. Linear regression analysis was conducted in Microsoft Excel, and the significance of multiple groups was analysed using SPSS Statistics 22 (IBM Corporation, Armonk, NY, USA).

## Supplementary Material

Web_Material_uhae215

## Data Availability

The data that support the findings are available within the article and supplementary data.
