# Supplementary material for: ABIOTIC STRESS GENE 1 mediates aroma volatiles accumulation by activating MdLOX1a in apple
Source: Hortic Res. 2024 Aug 8;11(10):uhae215. doi: 10.1093/hr/uhae215 (PMC11464680; doi:10.1093/hr/uhae215)
Supplement: Web_Material_uhae215 [file web_material_uhae215.zip › Supplemental figures.pdf]

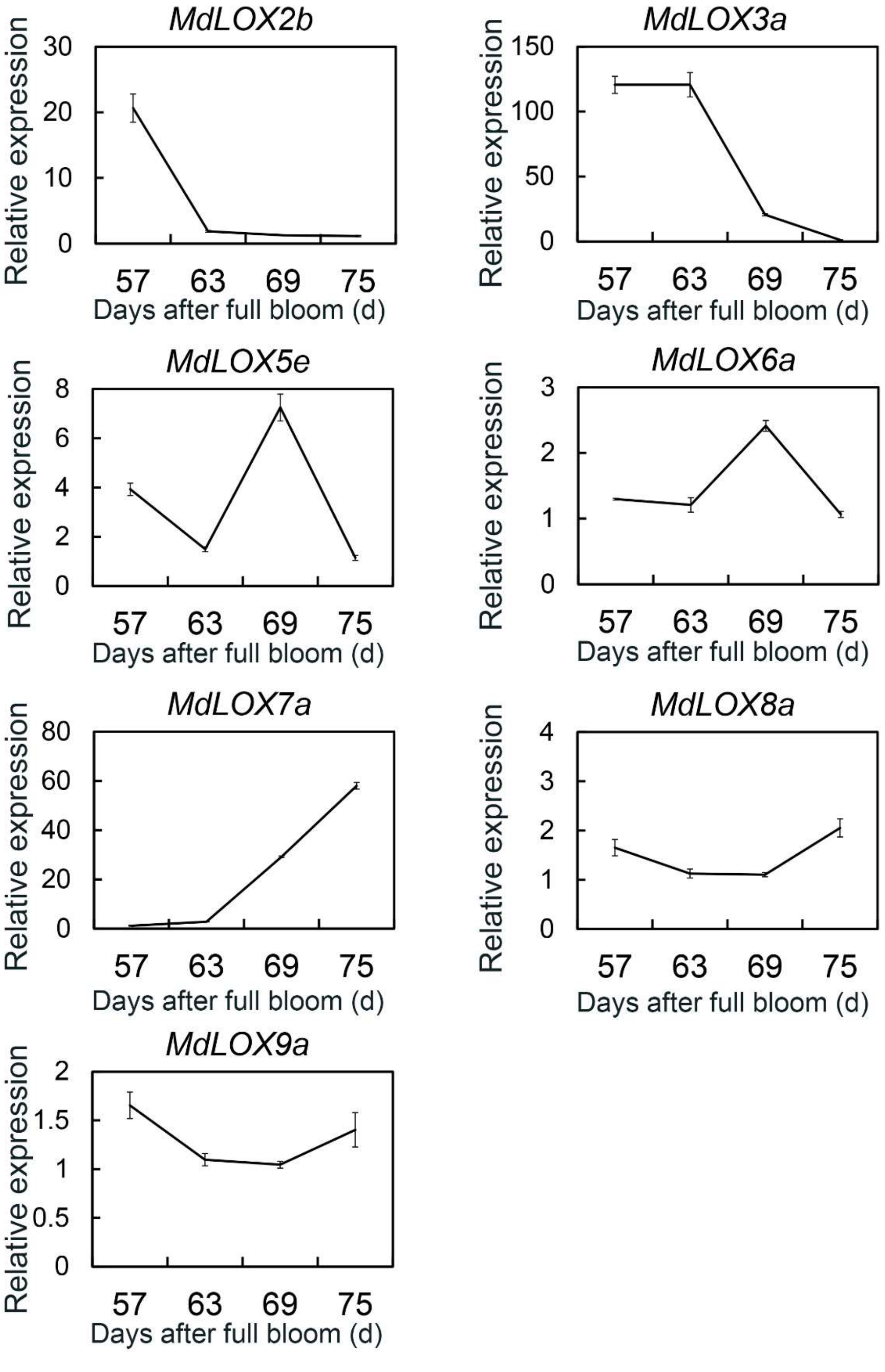

Figure S1. Relative expression of *MdLOX* genes during apple developmental stages. *MdActin* was used as an internal control gene. Error bars represent the standard deviation of three independent biological replicates.

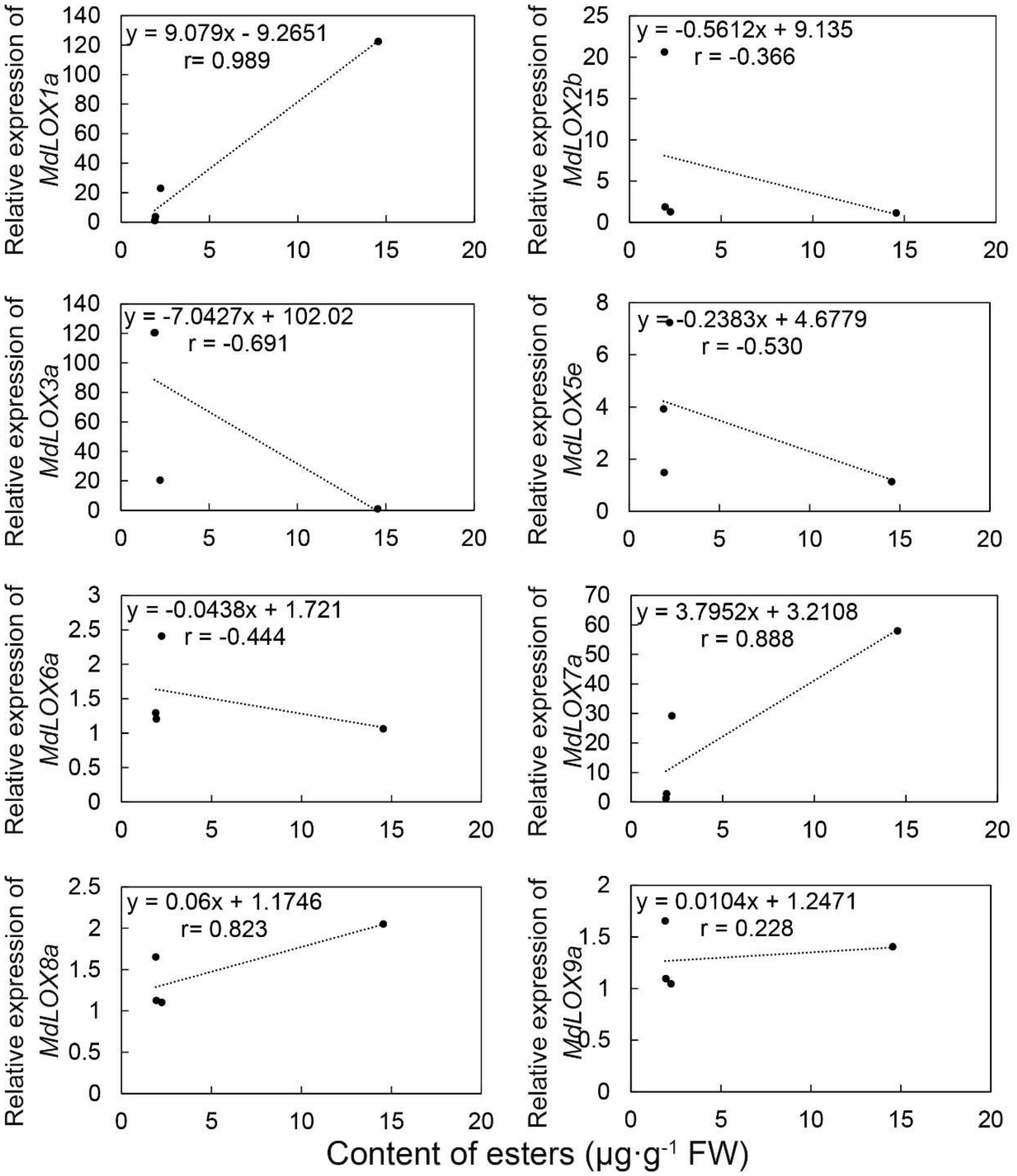

Figure S2. Analysis of linear regression between the ester content and *MdLOX* expression in apple fruit during the ripening stage. FW, Fresh weight.

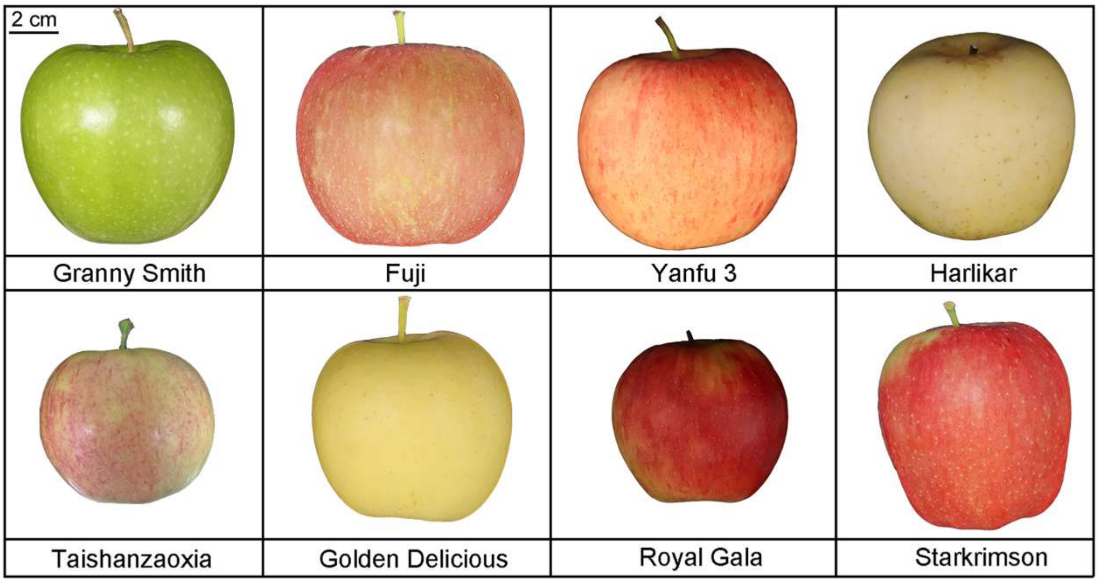

Figure S3. Fruit of eight apple cultivars harvested at ripening.

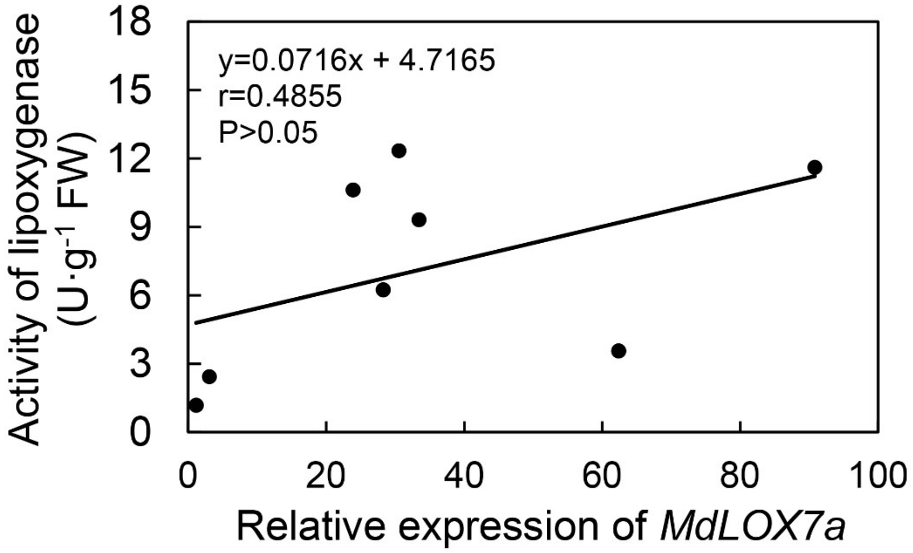

Figure S4. Analysis of linear regression between *MdLOX7a* expression and lipoxygenase activity in the fruit of eight apple cultivars. FW, Fresh weight. Significant differences were determined using Tukey one-way analysis of variance (ANOVA) with SPSS Statistics 22 (*$P < 0.05$ and **$P < 0.01$).

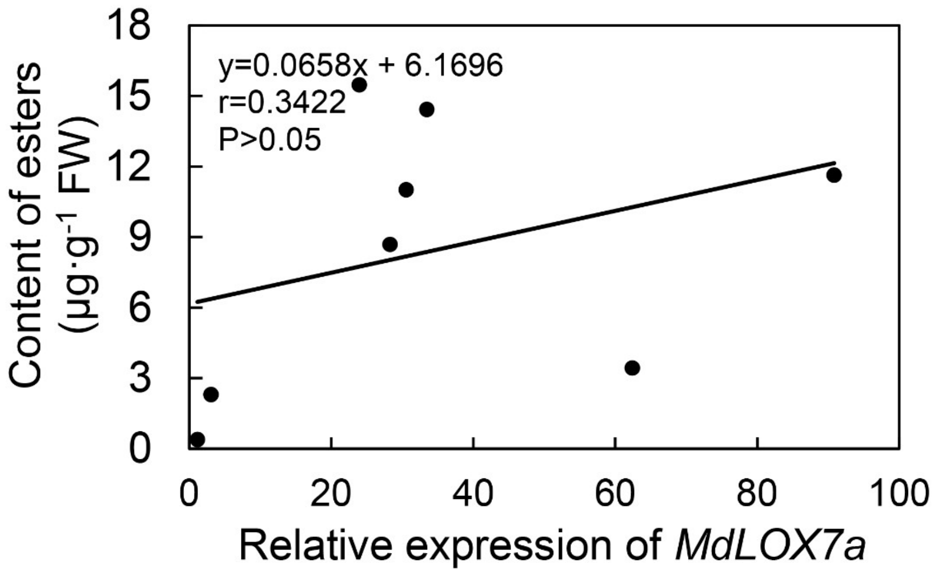

Figure S5. Analysis of linear regression between *MdLOX7a* expression and ester content in the fruit of eight apple cultivars. FW, Fresh weight. Significant differences were determined using Tukey one-way analysis of variance (ANOVA) with SPSS Statistics 22 (*P < 0.05 and **P < 0.01).

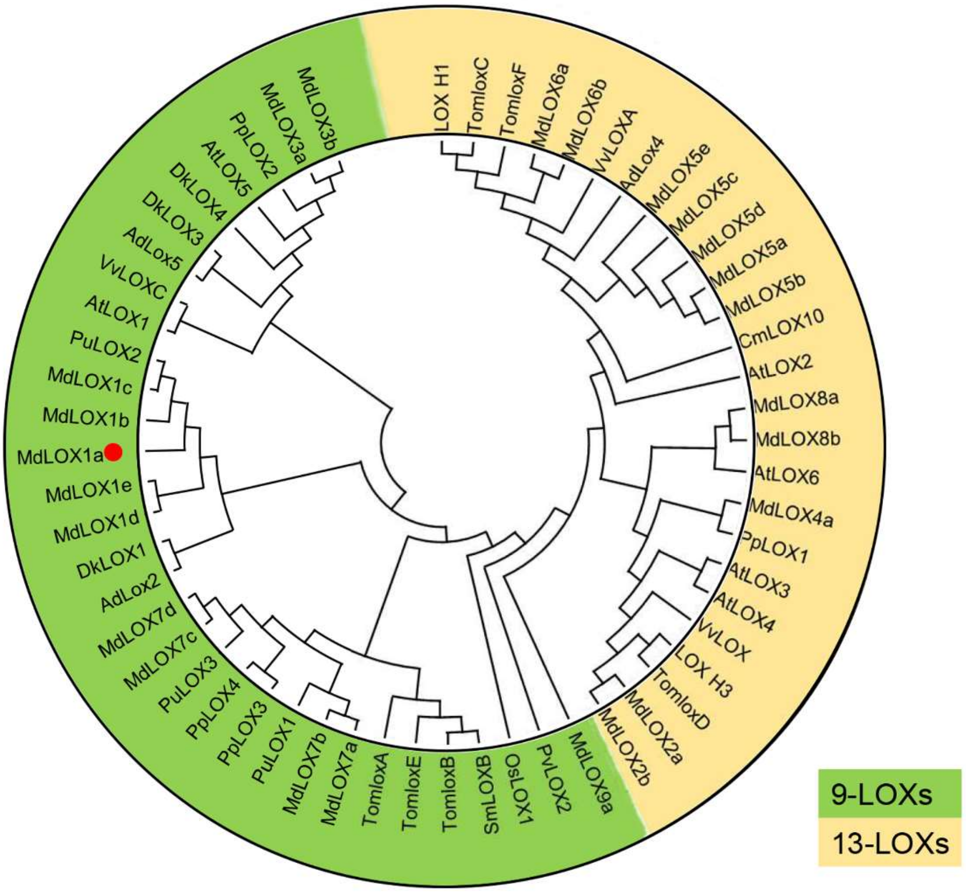

Figure S6. Phylogenetic instruction of plant LOX proteins. Phylogenetic analysis of 58 LOX proteins from 14 plant species. The LOX amino acid sequences were aligned with ClustalX and the phylogenetic tree was constructed using MEGA X.

```
MdLOX1a.seq    MLHNLLGKITGQQ..QDGESNIG.KINGTVVLMKKNVLDFNDFNASVLDRVHELVGQRVSLQLISAVHGDPDNGLKGNLG    77
MdLOX7a.seq    MLHSKYPTSSQDTTVDTQHKNEGKKIKGRVVLMKKVVLDVNDLKASILDRVDELLGKVVSLRLISSVNGDPENELKGKVG    80
Consensus      mlh                 n g ki g vvlmkk vld nd  as ldrv el g  vsl lis v gdp n lkg  g

MdLOX1a.seq    KQAYLEDWITTITPLTAGESAFKVTFDWEEEVGVPGAFIIQNNHHSEFFLKTVTLDNVPDEGRVHFVCNSWVYPAEKYTK   157
MdLOX7a.seq    KPAYLENWVTTIAPLTAGEAAFDVTFDWEKEIGVPGAFVVRNEHHSEFYLKTLTLEDVPGEGRVHFVCNSWVYPADKYKK   160
Consensus      k ayle w tti pltage af vtfdwe e gvpgaf   n hhsef lkt tl  vp egrvhfvcnswvypa ky k

MdLOX1a.seq    DRVFFTNKTYLPSEVPLPLRKYIEEELVELRGDGKRK..LEEWDRVYDYAYYNDLGDPDKGSEYVRPIMGGSTEYPYPRR   235
MdLOX7a.seq    DRVFFSNKTYISSDTPKPLQKFREEELVNLRGDDEERGELQEWDRVYDYAYYNDLGNPDKGPEYARPVLGGSIEYPYPRR   240
Consensus      drvff nkty  s  p pl k  eeelv lrgd      l ewdrvydyayyndlg pdkg ey rp  ggs eypyprr

MdLOX1a.seq    GRTGRPPKETDPNTESRLPIVSSLSIYVPRDERFGHLKMSDFLAYALKSIAQFIRPEIEALFDKTPNEFDSFKDVLQLYE   315
MdLOX7a.seq    GKTGRPATKTDPNTESRLKLIQILNVYVPRDERFGPIKMSDLLAYVLKSIPQVLKPEIRDLLVGNKDEFESMEEVLKLYE   320
Consensus      g tgrp   tdpntesrl     l  yvprderfg  kmsd lay lksi q   pei  l      ef s   vl lye

MdLOX1a.seq    GGIPLPEGLFKEIGDSIPAEMLKEIFRTDGAQFLRFPMPEVIKVDKTAWRTDEEFAREMLAGVNPVNIRLLQEFPPASKL   395
MdLOX7a.seq    GGLELPDGILKYISDSTPGEIFKELFRTDGEKFLKFPVPQVIKEDKSAWRTDEEFARELLAGVNPVTIRRLQEFPPSSEL   400
Consensus      gg  lp g  k i ds p e  ke frtdg  fl fp p vik dk awrtdeefare lagvnpv ir lqefpp s l

MdLOX1a.seq    DPKVYGDQTSTITEQHIKNNLDGLTVDEALKNKKLFILDHHDALMPYLRRINSTSNKIYGSRTLLFLKSDGTLKILVIEL   475
MdLOX7a.seq    DREAYGDQTSQITKEHIEHNLNGLTIDEAIMNNKLFILNHHDAWMPYLRRINTTSTKAYASRTLLFLNNDGTLKPIAIEL   480
Consensus      d   ygdqts it  hi  nl glt dea  n klfil hhda mpylrrin ts k y srtllfl  dgtlk   iel

MdLOX1a.seq    SLPHPDGDQYGCISNVYTPAEQGVESSIWQLAKAYVAVNDSGNHQLISHWLNTHAVIEPVIIAANRQLSVVHPIYKLLQP   555
MdLOX7a.seq    SLPHPDGDQFGCISKVYTPSSQGFESSLWQFAKGYVLVNDSGCHQLFSHWLRAHAVTEPFVIAANRQLSVLHPIHKLLHP   560
Consensus      slphpdgdq gcis vytp  qg ess wq ak yv vndsg hql shwl  hav ep  iaanrqlsv hpi kll p

MdLOX1a.seq    HFRDTMYINAIGRGILLNARGVIESTVFPARYALGLSSAVYKDWIFPEQALPADLIKRGVAVKDENSPHGLRLLIEDYPY   635
MdLOX7a.seq    HFRGTMNANASARQVLTNAGGVIEEIIFASKFSMEWSSAMYKDWTFPDQALPADLIKRGVAVEDSSASHGVRLLIEDYPY   640
Consensus      hfr tm  na  r  l na gvie   f        ssa ykdw fp qalpadlikrgvav d    hg rlliedypy

MdLOX1a.seq    AVDGIEIWFAIKTWVEDYCAFYYKTNEIIQTDVELQSWWKELVEEGHGDIKDEPWWPKMQTFEELVETCTILVWTASALH   715
MdLOX7a.seq    AADGLELWTAIKTWVKDYCSFYYKNDQMVQNDSELQSWWKELREVGHGDKNEEPWWPKMQTCDELIESCTTIIYIASAHH   720
Consensus      a dg e w aiktwv dyc fyyk     q d elqswwkel e ghgd   epwwpkmqt  el e ct     asa h

MdLOX1a.seq    AALNFGQFSYAGYLPNRPTISRKFMPEKGTPEYEELEASPDTVFLKTITAQLQTVLGIATIEILSRHSTDEVYLGQRDTP   795
MdLOX7a.seq    AAINYGQYSTGGFVPNRPTLSRRFMPEEGTPEYEELRTNPDKAFLKTFAPQLPTLLGMATVEILSRHPTDELYLGQRDTA   800
Consensus      aa n gq s  g  pnrpt sr fmpe gtpeyeel   pd  flkt   ql t lg at eilsrh tde ylgqrdt

MdLOX1a.seq    DWTSDTAALEAFEKFGKKLAEIEDRITSMNNDEKLKNRVGSVKIPYTLLFPTSEGGITGKGIPNSVS                862
MdLOX7a.seq    EWTTDADILQASEDFKKNLEAIEAKIEKMNKDERLKNRFGPAKIPYTLLYPSSEPGLTNKGVPTSIN                867
Consensus       wt d   l a e f k l  ie  i  mn de lknr g  kipytll p se g t kg p s
```

Sequence similarity=66.59%

Figure S7. Sequence alignment of MdLOX1a and MdLOX7a protein using DNAMAN software.

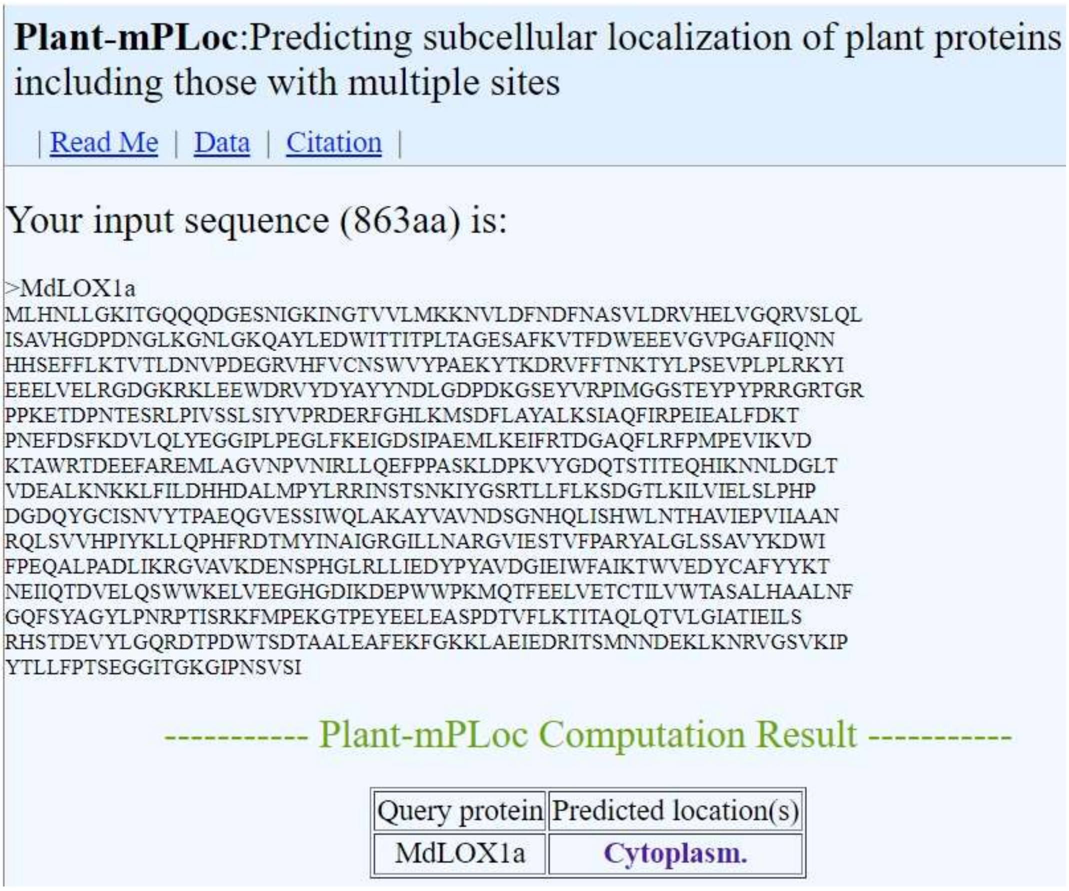

Plant-mPLoc:Predicting subcellular localization of plant proteins including those with multiple sites

| Read Me | Data | Citation |

Your input sequence (863aa) is:

>MdLOX1a
MLHNLLGKITGQQQDGESNIGKINGTVVLMKKNVLDFNDFNASVLDRVHELVGQRVSLQL
ISAVHGDPDNGLKGNLGKQAYLEDWITTITPLTAGESAFKVTFDWEEEVGVPGAFIIQNN
HHSEFFLKTVTLDNVPDEGRVHFVCNSWVYPAEKYTKDRVFFTNKTYLPSEVPLPLRKYI
EEELVELRGDGKRKLEEWDRVYDYAYYNDLGDPDKGSEYVRPIMGGSTEYPYPRRGRTGR
PPKETDPNTESRLPIVSSLSIYVPRDERFGHLKMSDFLAYALKSIAQFIRPEIEALFDKT
PNEFDSFKDVLQLYEGGIPLPEGLFKEIGDSIPAEMLKEIFRTDGAQFLRFPMPEVIKVD
KTAWRTDEEFAREMLAGVNPVNIRLLQEFPPASKLDPKVYGDQTSTITEQHIKNNLDGLT
VDEALKNKKLFILDHHDALMPYLRRINSTSNKIYGSRTLLFLKSDGTLKILVIELSLPHP
DGDQYGCISNVYTPAEQGVESSIWQLAKAYVAVNDSGNHQLISHWLNTHAVIEPVIIAAN
RQLSVVHPIYKLLQPHFRDTMYINAIGRGILLNARGVIESTVFPARYALGLSSAVYKDWI
FPEQALPADLIKRGVAVKDENSPHGLRLLIEDYPYAVDGIEIWFAIKTWVEDYCAFYYKT
NEIIQTDVELQSWWKELVEEGHGDIKDEPWWPKMQTFEELVETCTILVWTASALHAALNF
GQFSYAGYLPNRPTISRKFMPEKGTPEYEELEASPDTVFLKTITAQLQTVLGIATIEILS
RHSTDEVYLGQRDTPDWTSDTAALEAFEKFGKKLAEIEDRITSMNNDEKLKNRVGSVKIP
YTLLFPTSEGGITGKGIPNSVSI

----------- Plant-mPLoc Computation Result -----------

Query protein	Predicted location(s)
MdLOX1a	**Cytoplasm.**

Figure S8. Subcellular localization was predicted using Cell-PLoc 2.0.

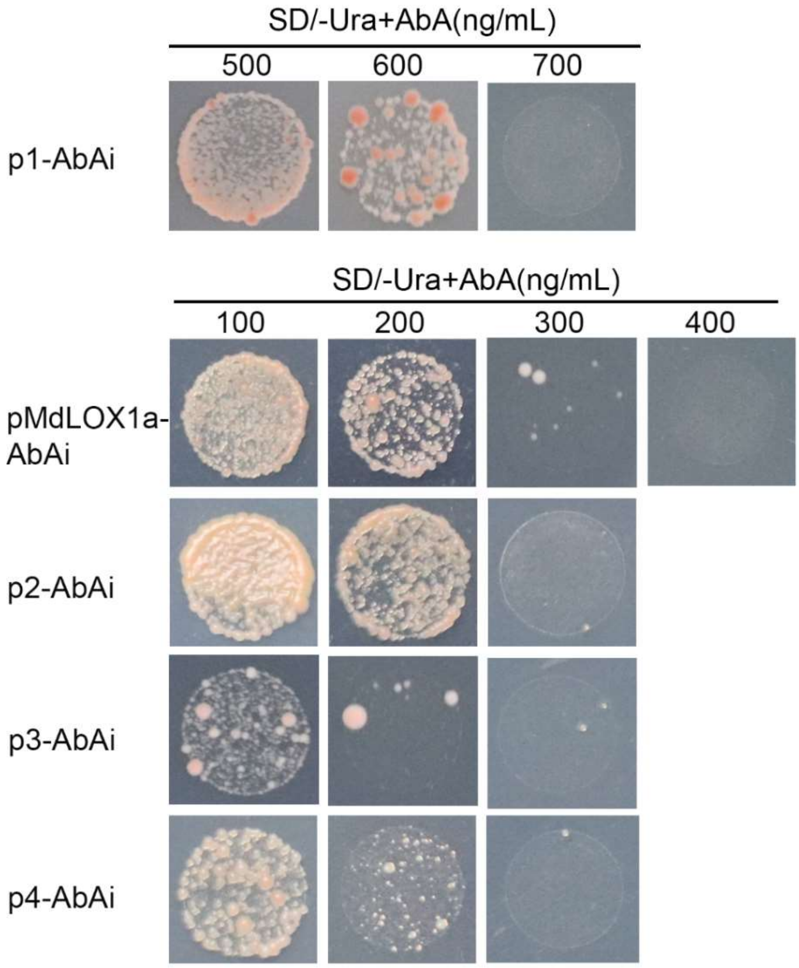

Figure S9. Background AbA^{r} expression in the yeast Y1H Gold strain containing specific promoters.

Sequence similarity=70%

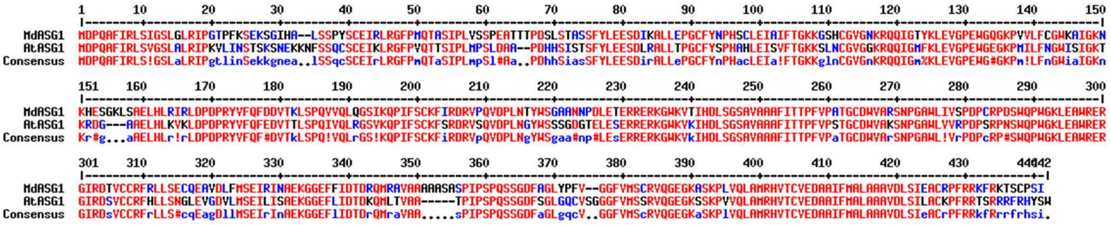

Sequence similarity=73%

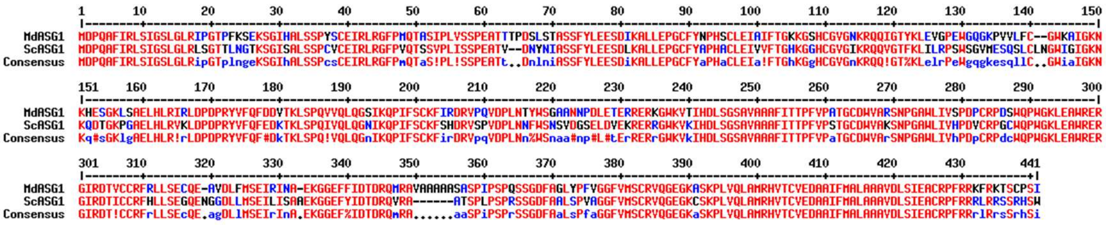

Figure S10. Protein sequence alignment of MdASG1 with AtASG1 and ScASG1.

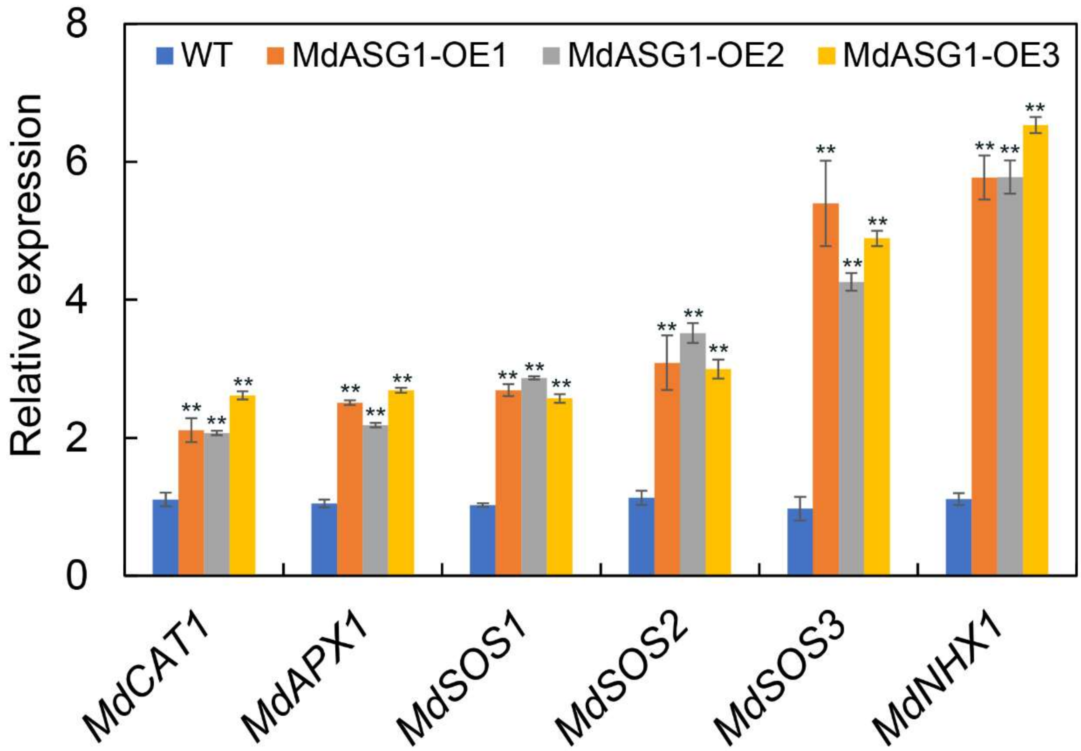

Figure S11. Relative expression of stress-related genes in apple calli of the WT and *MdASG1*-overexpressing transgenic lines (MdASG1-OE) in response to 50 mM NaCl treatment for 20 days. *MdActin* was used as an internal control gene. Error bars represent the standard deviation of three independent biological replicates. Significant differences were determined by two-sided Student's t-test. (**$P < 0.01$ and *$P < 0.05$).

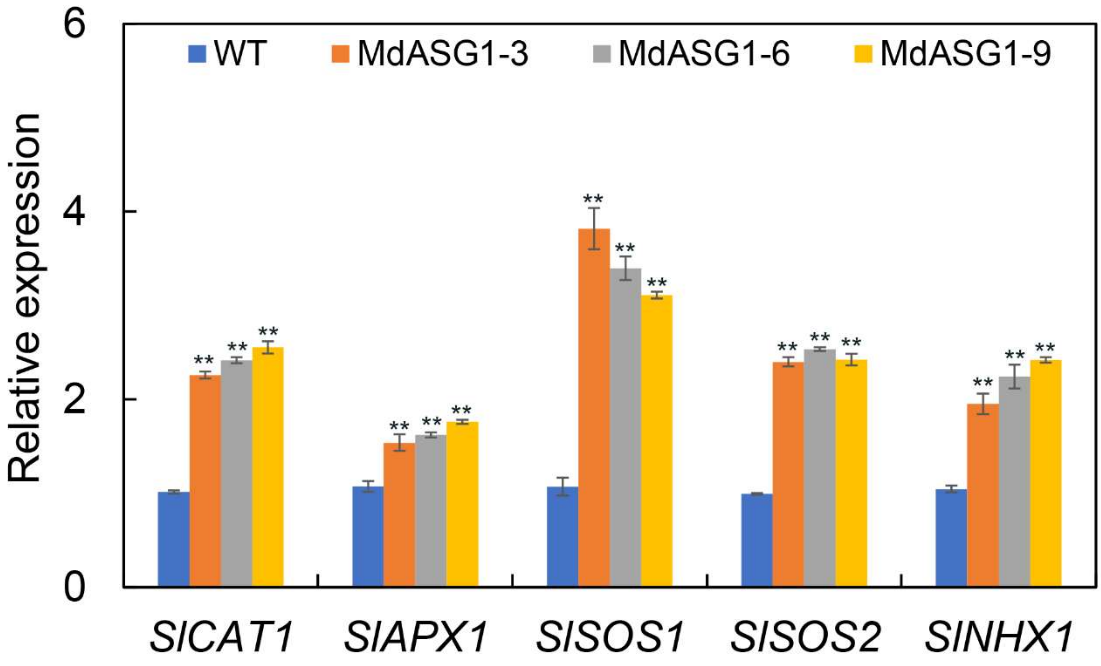

Figure S12. Relative expression of stress-related genes in the WT and *MdASG1*-overexpressing (MdASG1-3,6,9) tomato plants in response to 200 mM NaCl treatment for 20 days. *SlActin* was used as an internal control gene. Error bars represent the standard deviation of three independent biological replicates. Significant differences were determined by two-sided Student's t-test. (***P* < 0.01 and **P* < 0.05).

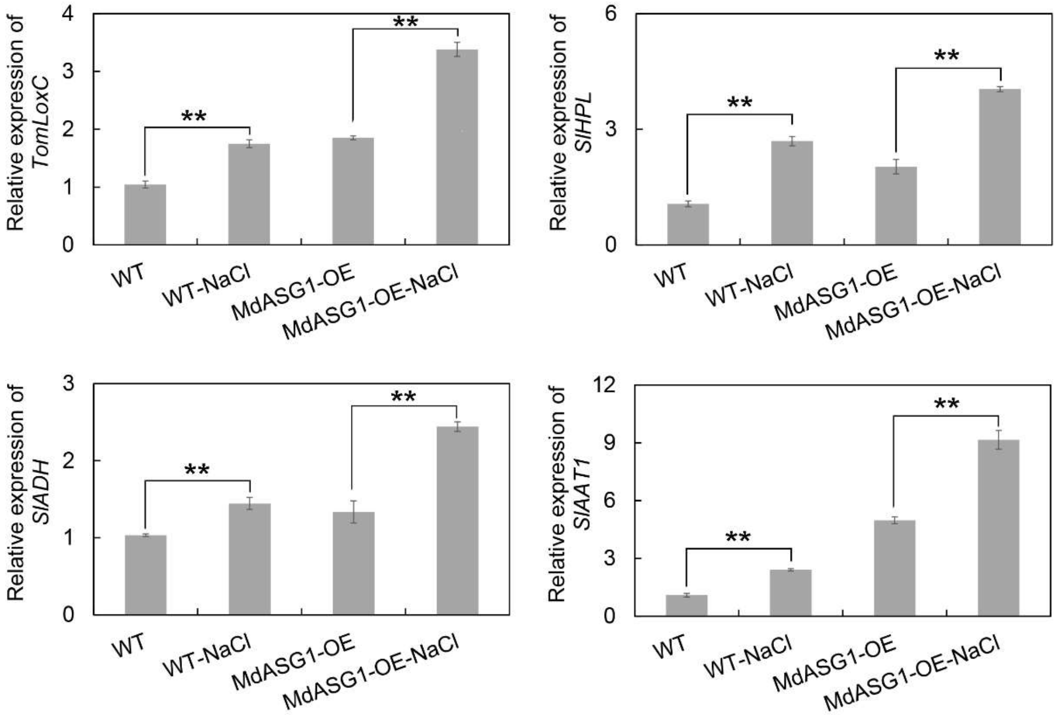

Figure S13. Transcriptional changes in fatty acid-derived volatile synthesis genes in response to 200 mM NaCl in ripening fruit of WT and *MdASG1*-overexpressing (MdASG1) tomato pants. *SlActin* was used as an internal control gene. Error bars represent the standard deviation of three independent biological replicates. Significant differences were determined by two-sided Student's t-test. (***P* < 0.01 and **P* < 0.05).

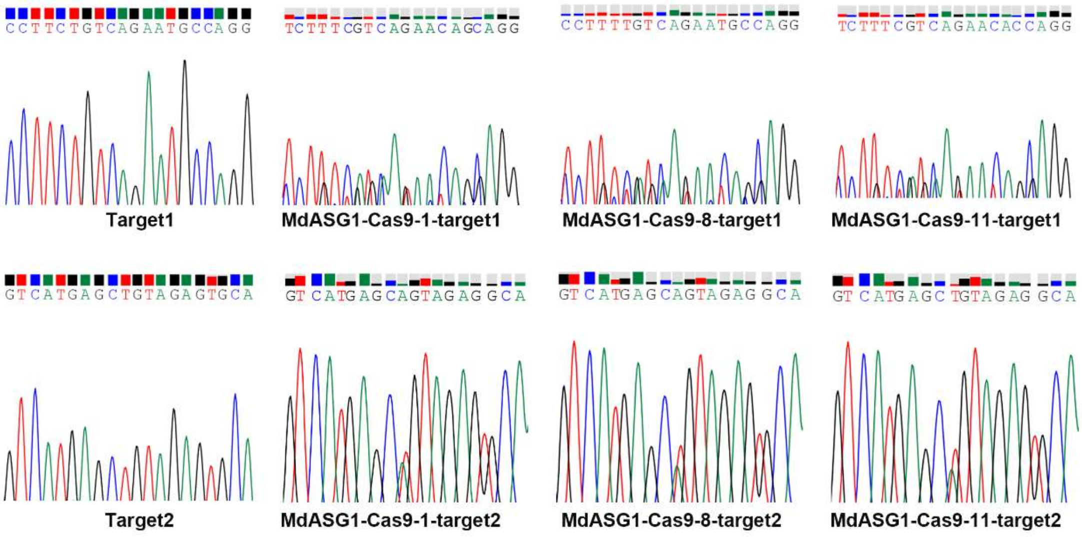

Figure S14. Peak chart of first-generation sequencing results of wild-type and *MdASG1*-Cas9 knockdown apple calli.
