# Supplementary material for: ABIOTIC STRESS GENE 1 mediates aroma volatiles accumulation by activating MdLOX1a in apple
Source: Hortic Res. 2024 Aug 8;11(10):uhae215. doi: 10.1093/hr/uhae215 (PMC11464680; doi:10.1093/hr/uhae215)
Supplement: Web_Material_uhae215 [file web_material_uhae215.zip › Supplemental tables.pdf]

Table S1. The content of apple ester aroma substances during fruit developmental stages.

Number	Compounds	Apple fruit ester aroma substance content (ug·g^{-1} FW)			
		**57 (DAFB)**	**63 (DAFB)**	**69 (DAFB)**	**75 (DAFB)**
1	Ethyl Acetate	N.D.	N.D.	N.D.	0.019526±0.004052 a
2	Methyl 2-methylbutanoate	N.D.	N.D.	N.D.	0.005443±0.000170 a
3	Ethyl butanoate	0.019406±0.000415 b	0.016318±0.000828 b	0.027499±0.000501 b	0.143479±0.032154 a
4	Butyl acetate	N.D.	0.000864±0.000091 b	0.002108±0.000125 b	0.199091±0.029848 a
5	Ethyl (E)-2-butenoate	N.D.	N.D.	0.001835±0.000052 b	0.002646±0.000043 a
6	Ethyl 2-methylbutanoate	N.D.	N.D.	0.018582±0.006041 b	0.049234±0.017082 a
7	1-Butanol, 2-methyl-, acetate	0.003563±0.000424 b	0.008131±0.000980 b	0.007862±0.000075 b	0.066675±0.003524 a
8	Propyl butanoate	N.D.	N.D.	N.D.	0.016625±0.012385 a
9	Ethyl pentanoate	0.001857±0.000121 b	0.002704±0.000729 b	0.004341±0.000768 a	0.005072±0.000302 a
10	Butyl propionate	N.D.	N.D.	N.D.	0.017746±0.006560 a
11	Pentyl acetate	0.003144±0.000451 b	N.D.	N.D.	0.027542±0.000939 a
12	Methyl hexanoate	N.D.	N.D.	0.003401±0.000556 b	0.008850±0.000528 a
13	Ethyl tiglate	0.001787±0.000103 a	N.D.	0.008050±0.004448 a	0.003607±0.000047 a
14	Propyl 2-methylbutanoate	N.D.	N.D.	N.D.	0.006306±0.005424 a
15	Butyl 2-methylpropanoate	N.D.	N.D.	N.D.	0.007875±0.001635 a
16	2-Methylpropyl butyrate	N.D.	N.D.	N.D.	0.005386±0.002614 a
17	Ethy 4-methypentanoate	0.001855±0.000131 a	0.001863±0.000083 a	0.002624±0.000713 a	N.D.
18	Butyl butyrate	N.D.	0.001444±0.000638 b	N.D.	0.810190±0.090530 a
19	Ethyl hexanoate	0.117268±0.010703 b	0.104966±0.008617 b	0.123895±0.040940 b	0.405922±0.081709 a

Number	Compounds	Apple fruit ester aroma substance content (ug·g^{-1} FW)			
		57 (DAFB)	63 (DAFB)	69 (DAFB)	75 (DAFB)
20	2-Methylpropyl 2-methylbutanoate	N.D.	N.D.	N.D.	0.014679±0.000642 a
21	3-Hexen-1-ol, acetate, (Z)-	0.054988±0.004170 a	0.053115±0.002216 a	0.059908±0.001435 a	0.025712±0.010776 b
22	4-Hexenyl propionate	N.D.	N.D.	N.D.	0.011201±0.000472 a
23	3-Hexen-1-ol, propanoate, (Z)-	N.D.	N.D.	N.D.	0.004537±0.000472 a
24	Ethyl (Z)-3-hexenoate	0.005022±0.000217 b	N.D.	0.007936±0.000637 a	0.005311±0.001575 b
25	Hexyl acetate	0.669920±0.033550 b	0.732295±0.031896 b	0.754730±0.025363 b	1.251860±0.122608 a
26	2-Hexen-1-ol, acetate, (Z)-	0.836976±0.041904 ab	0.741858±0.027845 b	0.850643±0.028167 a	0.529971±0.060267 c
27	Butyl 2-methylbutanoate	N.D.	0.000883±0.000071 b	N.D.	0.553273±0.099993 a
28	2-Methylbutyl butanoate	N.D.	N.D.	N.D.	0.026190±0.007473 a
29	Ethyl (E)-4-heptenoate	0.002299±0.000059 b	N.D.	0.007309±0.000524 a	N.D.
30	Pentyl butanoate	N.D.	N.D.	N.D.	0.155718±0.027306 a
31	Ethyl heptanoate	N.D.	0.003549±0.000403 b	0.003095±0.001688 b	0.018685±0.013717 a
32	2-Methylbutyl pentanoate	N.D.	N.D.	N.D.	0.022080±0.000943 a
33	Hexyl propionate	0.006443±0.000206 b	0.011776±0.001414 b	0.004876±0.002154 b	0.163452±0.028701 a
34	2-Hexen-1-ol, propanoate, (E)-	0.008676±0.001102 b	0.012341±0.001568 b	0.005711±0.001228 b	0.021917±0.011643 a
35	Heptyl acetate	N.D.	N.D.	N.D.	0.007530±0.001314 a
36	n-Butyl tiglate	N.D.	N.D.	N.D.	0.019942±0.003032 a
37	Pentyl 2-methylbutanoate	N.D.	N.D.	N.D.	0.103890±0.004551 a
38	Hexyl 2-methylpropanoate	0.002261±0.000400 b	0.002541±0.000407 b	0.002087±0.000883 b	0.111852±0.004711 a
39	trans-2-Hexenyl isobutyrate	0.003894±0.000352 a	0.003541±0.000240 a	N.D.	N.D.

Number	Compounds	Apple fruit ester aroma substance content (ug·g^{-1} FW)			
		57 (DAFB)	63 (DAFB)	69 (DAFB)	75 (DAFB)
40	2-Ethylhexyl-2-methypropanoate	N.D.	N.D.	0.005143±0.001375 a	N.D.
41	2-Ethylhexyl hexenoate	N.D.	N.D.	N.D.	0.016120±0.001690 a
42	Ethyl benzoate	N.D.	N.D.	0.002530±0.001701 a	N.D.
43	3-Hexenyl butanoate	0.003498±0.000281 b	0.005180±0.000782 b	N.D.	0.016520±0.001853 a
44	Hexyl butanoate	0.048835±0.003313 b	0.094601±0.028256 b	0.082403±0.001853 b	3.599587±0.347999 a
45	2-Hexenyl butanoate	0.080868±0.005558 b	0.105665±0.019697 b	0.055889±0.001938 b	0.225708±0.154022 a
46	3-Methylpentyl acetate	N.D.	N.D.	N.D.	0.014553±0.000825 a
47	5-Hexenyl pentanoate	N.D.	N.D.	N.D.	0.012761±0.001303 a
48	trans-2-Hexenyl 2-methylbutyrate	0.006315±0.001261 a	N.D.	N.D.	N.D.
49	cis-3-Hexenyl-.alpha.-methylbutyrate	N.D.	N.D.	N.D.	0.025835±0.007426 a
50	Hexyl 2-methyl butyrate	N.D.	0.004459±0.000432 b	N.D.	4.072193±0.238707 a
51	trans-2-Hexenyl 2-methylbutyrate	N.D.	0.007707±0.001322 a	N.D.	N.D.
52	Hexyl 2-butenoate	N.D.	N.D.	N.D.	0.035661±0.000709 a
53	Ethyl (E)-2-octenoate	N.D.	N.D.	N.D.	0.006150±0.002790 a
54	(3Z)-3-Hexenyl (2E)-2-butenoate	N.D.	N.D.	N.D.	0.006995±0.000920 a
55	2-Methylbutyl hexanoate	N.D.	N.D.	N.D.	0.058246±0.009056 a
56	Pentyl hexanoate	N.D.	N.D.	N.D.	0.159566±0.018353 a
57	4-methylpentyl 2-methylcrotonate	N.D.	N.D.	N.D.	0.009318±0.003338 a
58	Hexyl tiglate	N.D.	N.D.	N.D.	0.097147±0.011629 a
59	Heptyl 2-methylbutanoate	N.D.	N.D.	N.D.	0.011741±0.000433 a

Number	Compounds	Apple fruit ester aroma substance content (ug·g^{-1} FW)			
		57 (DAFB)	63 (DAFB)	69 (DAFB)	75 (DAFB)
60	Butyl hexyl carbonate	N.D.	N.D.	N.D.	0.011637±0.007883 a
61	Hexyl hexanoate	0.002804±0.000212 b	0.004051±0.000781 b	0.002010±0.000325 b	1.327865±0.002885 a
62	2-Hexenyl (E)-hexanoate	0.005049±0.000371 a	0.005672±0.000269 a	N.D.	N.D.
63	Octyl 2-methylbutyrate	N.D.	N.D.	N.D.	0.012257±0.001301 a
64	Diethyl Phthalate	0.002747±0.000853 b	0.002673±0.000385 b	0.018219±0.000501 a	0.026146±0.010316 a
65	Isobutyl phthalate	0.011467±0.003074 a	0.009954±0.001531 a	0.036619±0.020028 a	N.D.
66	Dibutyl phthalate	N.D.	N.D.	N.D.	0.029032±0.008868 a

Note: DAFB represents days after full bloom. Different letters represent significant difference according to the Duncan's new multiple range test (P < 0.05). N.D. indicates that the composition is not detected. Error bars, mean ± SD, 3 biological replicates.

Table S2 The content of aldehydes, alcohols and esters in ‘Orin’ calli.

Number	Compounds	Aroma substance content in calli (μg·g^{-1} FW)			
		WT	MdLOX1a-OE1	MdLOX1a-OE2	MdLOX1a-OE3
	**Aldehydes**				
1	Hexanal	0.012573±0.000584 a	0.012073±0.001976 a	0.011239±0.001667 a	0.012361±0.000804 a
2	2-Ethylhexanal	N.D.	0.013205±0.002289 a	0.010836±0.001201 a	0.013961±0.000325 a
3	Nonanal	0.040961±0.002600 b	0.086856±0.014661 a	0.059373±0.003833 b	0.099922±0.007171 a
4	5-Octadecenal	N.D.	0.007277±0.000377 a	0.006452±0.000808 b	0.007845±0.000124 a
	**Alcohols**				
5	1,5-Hexadien-3-ol	N.D.	0.062145±0.004190 a	0.073343±0.009744 a	0.054486±0.001001 a
6	1-Penten-3-ol	0.048284±0.005247c	0.072432±0.001247 a	0.075090±0.002939 ab	0.051932±0.001327 b
7	3-Methyl-1-butanol	0.050407±0.005436 a	0.038748±0.000816 b	0.039657±0.001528 b	0.035524±0.000820 b
8	1-Hexanol	0.054325±0.003847 c	0.080311±0.000948 a	0.075209±0.006794 ab	0.067595±0.000839 b
9	2-Ethyl-1-hexanol	0.042630±0.005249 b	0.073166±0.004752 a	0.077826±0.001281 a	0.067558±0.002082 a
10	2-Propyl-1-pentanol	N.D.	0.024890±0.003718 b	0.039449±0.000881 a	0.025349±0.002700 b
11	3-Nonanol	0.243361±0.020822 c	0.310402±0.017003 b	0.407649±0.012802 a	0.238865±0.012332 c
12	3-Nonen-1-ol, (Z)-	N.D.	0.014420±0.000471 b	0.035753±0.000472 a	0.013837±0.000549 b
	**Esters**				
13	Ethyl 2-methylbutanoate	0.008007±0.000967 c	0.031707±0.004028 b	0.048509±0.000945 a	0.030322±0.005218 b
14	Ethyl heptanoate	0.018882±0.001706 c	0.028460±0.003379 bc	0.063800±0.007748 a	0.033885±0.005859 b
15	2-Octanol, acetate	0.004567±0.000050 c	0.030007±0.002693 b	0.050008±0.002698 a	0.029946±0.002646 b
16	Diethyl Phthalate	0.023692±0.003875 c	0.055141±0.003471 b	0.077771±0.007689 a	0.055424±0.003438 b

Number	Compounds	Aroma substance content in calli (μg·g^{-1} FW)			
		**WT**	**MdLOX1a-OE1**	**MdLOX1a-OE2**	**MdLOX1a-OE3**
17	Isopropyl myristate	0.079128±0.005321 b	0.089823±0.006445 b	0.106524±0.006184 a	0.089686±0.006453 b

Note: Different letters represent significant difference according to the Duncan's new multiple range test (P < 0.05). N.D. indicates that the composition is not detected. Error bars, mean ± SD, 3 biological replicates.

Table S3. Primes were employed in this research for RT-qPCR analysis.

**Name**	**Forward primer (5′-3′)**	**Reverse primer (5′-3′)**
MdLOX1a	TCGGAAAGAAACTGGCTGAAAT	TAGATAGAGACGCTGTTGGGAAT
MdLOX2b	CCAATACAGGACCAAGTTCAAGAT	AACTGTCTATGTGCTGCCAAG
MdLOX3a	GGTCCAAGGTGATTCTGAACT	CGTAAGAGTATTGCCCGAAATTG
MdLOX5e	GAACCAACATGCCAACTGAAG	ACACATCTAAGACAGCCATCAC
MdLOX6a	GACCAATGTGCCCACTGAG	ACGCCTGCTCCATCTTCT
MdLOX7a	GGACAACGGATGCAGACATAC	CCAGGCTCACTAGAAGGATACA
MdLOX8a	AACCGTCCGACCATTATGAG	GTGAGTTGAGAGCGTATCTTGTA
MdLOX9a	GTGATAAGAGCAGCGAGACAT	AATGAACCTTCGGCACAGT
MdASG1	TCATAGACACAGACAGACAGA	GACGAAACCACCCACAAA
MdHPL	GTTGCTCTGTGGGTTTCA	CTGCTTGTTGGACTCACTT
MdADH	GGGTGTTGCTGTTCTTGT	GCTCCAGTTCCTTGTTCAT
MdAAT1	CTCATACGCATCCAGTAACC	CGACATTTCCACAAACAAGC
MdCAT1	GGAACAATATGCCCGTCTTCTT	GTGCCTGTAGTTGAGTGATACC
MdAPX1	CACAAGGAGCGTTCTGGATT	ACAAGAGGGCGGAAGACA
MdSOS1	GCAGTATGGTGAATGTGAAT	CGTGATATGATGGAACAGTAG
MdSOS2	ACCATTCTCAAGCACAGA	ACAATTCTCCTCCAGTTACA
MdSOS3	ACAGGGTACATTGAGCGAGAGGAG	TCATCAATCCGCCCATCACCTTTC
MdNHX1	GCTTACAATCAGTTTACG	GAATGAGGCAGCAAGAAC
MdASG1-C	CGGTCCCTTCTGTCAGAATG	CCTTCCCCTTGCACTCTACA
MdActin	TGACCGAATGAGCAAGGAAATTACT	TACTCAGCTTTGGCAATCCACATC
TomloxC	GCATCCTCATTTCAGATACAC	TCTCCATTCAGCACCATAG
TomloxD	GGCTTGCTTTACTCCTGGTC	AAATCAAAGCGCCAGTTCTT

**Name**	**Forward primer (5′-3′)**	**Reverse primer (5′-3′)**
SlHPL	CACAGAACAAGAAGCCATT	GGATTTACGCCGACTTTG
SlADH	AGGCATGGTCTGGTGGAATA	GGGAGAGTGATGGAAGGGTAA
SlAAT1	CAATAAATTACCACAAGCCAAAAC	AACATTAGTATGGGGATTTGGAG
SlCAT1	TCCCAGTTAATGCTCCCAAGT	CGCCTTCCATTCAAGACACAA
SlAPX1	GGCTGTGACCACTTGAGAG	GCTCCGTGAAGTATGAATTGTC
SlSOS1	GCGAGGAAGAAGGAGAGGAT	GTCTGGACAGCATCGTGAAG
SlSOS2	GTCAGGATTATGTCAAGCGTCAA	CAGCCCTATTTGCCGTTACC
SlNHX1	ACCAGCACAATTACAATAGTCCTC	GGATCAACATTTCCCATCTCAAGT
SlASG1	GCTGTCGCTTCCATCTTCTAT	CTTGGACTAGGCAATGGACTC
SlActin	TGGTCGGAATGGGACAGAAG	CTCAGTCAGGAGAACAGGGT

Table S4 The genes ID were employed in this research.

**Gene**	**ID**
MdHPL	LOC103427164
MdADH	LOC103428551
MdAAT1	KC291133.1
MdCAT1	GU321226.1
MdAPX1	EF528482.1
MdSOS1	XM_050273660.1
MdSOS2	XP_008362901.2
MdSOS3	XM_029106206.1
MdNHX1	GU338395.1
MdActin	MZ605395.1
TomloxC	NM_001246883.2
TomloxD	U37840.2
SlHPL	NM_001247491.2
SlADH	EF613492.1
SlAAT1	DQ099420.1
SlCAT1	M93719.1
SlAPX1	DQ099420.1
SlSOS1	AJ717346.1
SlSOS2	AJ717348.1
SlNHX1	AJ306630.1
SlASG1	LOC101261157
SlActin	AB199316.1
AtASG1	AT5G17640.1

Table S5 Accession numbers for LOXs in phylogenetic analysis.

**Name**	**Species**	**Accession**
AdLox2	*Actinidia deliciosa*	DQ497797
AdLox4	*Actinidia deliciosa*	DQ497793
AdLox5	*Actinidia deliciosa*	DQ497796
AtLOX1	*Arabidopsis thaliana*	NM_104376
AtLOX2	*Arabidopsis thaliana*	NP_566875
AtLOX3	*Arabidopsis thaliana*	NP_564021
AtLOX4	*Arabidopsis thaliana*	AT1G72520
AtLOX5	*Arabidopsis thaliana*	AT3G22400
AtLOX6	*Arabidopsis thaliana*	NP_176923
CmLOX10	*Cucumis melo*	KT613843
DkLOX1	*Diospyros kaki*	JF436951
DkLOX3	*Diospyros kaki*	KF035131
DkLOX4	*Diospyros kaki*	KF035132
LOX H1	*Solanum tuberosum*	X96405
LOX H3	*Solanum tuberosum*	X96406
MdLOX1a	*Malus domestica*	KC489090
MdLOX1b	*Malus domestica*	MDP0000312397
MdLOX1c	*Malus domestica*	MDP0000423544
MdLOX1d	*Malus domestica*	MDP0000146677
MdLOX1e	*Malus domestica*	MDP0000312394
MdLOX2a	*Malus domestica*	KC494391
MdLOX2b	*Malus domestica*	KC494386
MdLOX3a	*Malus domestica*	KC747491
MdLOX3b	*Malus domestica*	MDP0000204470
MdLOX4a	*Malus domestica*	MDP0000224150
MdLOX5a	*Malus domestica*	MDP0000169311
MdLOX5b	*Malus domestica*	MDP0000753547
MdLOX5c	*Malus domestica*	MDP0000279287
MdLOX5d	*Malus domestica*	MDP0000174168
MdLOX5e	*Malus domestica*	MDP0000211556
MdLOX6a	*Malus domestica*	KC706492

**Name**	**Species**	**Accession**
MdLOX6b	*Malus domestica*	MDP0000300321
MdLOX7a	*Malus domestica*	KC494384
MdLOX7b	*Malus domestica*	MDP0000125303
MdLOX7c	*Malus domestica*	MDP0000172092
MdLOX7d	*Malus domestica*	MDP0000923670
MdLOX8a	*Malus domestica*	KC494378
MdLOX8b	*Malus domestica*	KC494377
MdLOX9a	*Malus domestica*	KC494373
OsLOX1	*Oryza sativa*	DQ389164
PpLOX1	*Prunus persica*	EU883638
PpLOX2	*Prunus persica*	FJ029110
PpLOX3	*Prunus persica*	FJ032015
PpLOX4	*Prunus persica*	EF568783
PuLOX1	*Pyrus ussuriensis*	EF215448
PuLOX2	*Pyrus ussuriensis*	EF215449
PuLOX3	*Pyrus ussuriensis*	EF215450
PvLOX2	*Phaseolus vulgaris*	U76687
SmLOXB	*Solanum muricatum*	KY783403
TomloxA	*Lycopersicon esculentumm*	U09026
TomloxB	Lycopersicon esculentumm	U09025
TomloxC	*Lycopersicon esculentumm*	U37839
TomloxD	*Solanum lycopersicum*	NM_001320292
TomloxE	*Solanum lycopersicum*	NM_001247169
TomloxF	*Solanum lycopersicum*	NM_001247330
VvLOXA	*Vitis vinifera*	NM_001281094
VvLOXC	*Vitis vinifera*	NM_001281249
VvLOXO	*Vitis vinifera*	XM_002273222

Table S6. Primers were employed to construct or verified the vectors in this research.

Name	Vectors	Forward primer (5′-3′)	Reverse primer (5′-3′)
MdLOX1a	PRI101	ttgatacatatgcccgtcgacATGTTGCACAACCTGCTTGGC	ttcggatccggtacccccgggGATAGAGACGCTGTTGGGAATTCC
	pTRV2	gtgagtaaggttaccgaattcCTCAACTTCGGACAGTTCTC	gagacgcgtgagctcggtaccAGTGTACGGAATCTTGACTG
	pHB	accagtctctctctcaagcttATGTTGCACAACCTGCTTGGC	gcccttgctcaccatggatccGATAGAGACACTGTTGGGAATTCCC
MdASG1	pGADT7	gtaccagattacgctcatatgATGGATCCTCAGGCTTTTATTAGG	cagctcgagctcgatggatccTCATATAGAAGGGCAGGAGGTTTT
	PCB302	ctccccttgctccgtggatccATGGATCCTCAGGCTTTTATTAGG	aacgtcgtatgggtaaggcctTATAGAAGGGCAGGAGGTTTTCC
	pGreenII62-SK	cgctctagaactagtggatccATGGATCCTCAGGCTTTTATTAGG	gtcgacggtatcgataagcttTATAGAAGGGCAGGAGGTTTTCC
	pHB	accagtctctctctcaagcttATGGATCCTCAGGCTTTTATTAGG	gcccttgctcaccatggatccTATAGAAGGGCAGGAGGTTTTCC
	PRI101	ttgatacatatgcccgtcgacATGGATCCTCAGGCTTTTATTAGG	ttcggatccggtacccccgggTATAGAAGGGCAGGAGGTTTTCC
	pGEX-4T	gatctggttccgcgtggatccATGGATCCTCAGGCTTTTATTAGG	ctcgagtcgacccgggaattcTCATATAGAAGGGCAGGAGGTTTT
	pTRV2	gtgagtaaggttaccgaattcGAATGCCAGGAGGCCGTG	gagacgcgtgagctcggtaccAGGGCAGGAGGTTTTCCTGA
	PCBC-DT1DT2	ATATATGGTCTCGATTGCCTTCTGTCAGAATGCCAGGGTT	ATTATTGGTCTCGAAACTGCACTCTACAGCTCATGACCAA
		TGCCTTCTGTCAGAATGCCAGGGTTTTAGAGCTAGAAATAGC	AACTGCACTCTACAGCTCATGACCAATCTCTTAGTCGACTCTAC
pMdLOX1a	pAbAi	gaaaagcttgaattcgagctcTCGGGAGGAAAAATCGGG	agcacatgcctcgaggtcgacTGTGTTCTAGTTTCCAAGAACTAATTAAG
	pGreenII0800-Luc	gggccccccctcgaggtcgacTCGGGAGGAAAAATCGGG	cgctctagaactagtggatccTGTGTTCTAGTTTCCAAGAACTAATTAAGC
p1MdLOX1a	pAbAi	gaaaagcttgaattcgagctcTCGGGAGGAAAAATCGGG	agcacatgcctcgaggtcgacTTGTAGTAAATCCATCATTTGCAAATT

Name	Vectors	Forward primer (5′-3′)	Reverse primer (5′-3′)
p2MdLOX1a	pAbAi	gaaaagcttgaattcgagctcGTTCATCCAATACTTATGGAAATAGTGTAC	agcacatgcctcgaggtcgacAAGAGATGCATGTACGGCTGAA
p3MdLOX1a	pAbAi	gaaaagcttgaattcgagctcTCTTTTTATTGTGAATAAACTAATCCCA	agcacatgcctcgaggtcgacTTAATTTCTGTCAGTTGATCTTCTTCAA
p4MdLOX1a	pAbAi	gaaaagcttgaattcgagctcCAAGGAGTGTGTGGATAGCACACC	agcacatgcctcgaggtcgacTGTGTTCTAGTTTCCAAGAACTAATTAAGC
35S	PRI101	ACTATCCTTCGCAAGACCCTTCCTCTAT	
GFP-F	pHB	CATTTCATTTGGAGAGGACACG	
TRV1	pTRV1	TTACAGGTTATTTGGGCTAG	CCGGGTTCAATTCCTTATC
TRV2	pTRV2	ACATTGTTACTCAAGGAAGCACG	AAGATCAGTCGAGAATGTCAATCTC
188F	PCB302	CCTCTCACCTTTTCGCTGTAC	

Table S7. Probes were employed for EMSA in this research.

Name	Forward primer (5′-3′)	Reverse primer (5′-3′)
p4MdLOX1<br>*a-1*	CAAGGAGTGTGTGGATAGCACACCCTGTGAGGAAATTGGCAC<br>TTTTCACATTATAAAACATAAATT	AATTTATGTTTTATAATGTGAAAAGTGCCAATTTCCTCACAGGG<br>TGTGCTATCCACACACTCCTTG
p4MdLOX1<br>*a-2*	AATTACGCAGGGATTACATCTATGTCATCTTAAAGCCAGCTAA<br>CTTTCGGGATAAACGATTTTGTAC	GTACAAAATCGTTTATCCCGAAAGTTAGCTGGCTTTAAGATGAC<br>ATAGATGTAATCCCTGCGTAATT
p4MdLOX1<br>*a-3*	GACTTTTTCACGTTTTCCCCTTTAAATACGACCACCCTTCATTG<br>AACTCTTCCATGCAAAGTTTCGA	TCGAAACTTTGCATGGAAGAGTTCAATGAAGGGTGGTCGTATTT<br>AAAGGGGAAAACGTGAAAAAGTC
p4MdLOX1<br>*a-4*	CTTTCAATTTATTTCACAATTATTCACAACATTCTTTGCTTAAT<br>TAGTTCTTGGAAACTAGAACACA	TGTGTTCTAGTTTCCAAGAACTAATTAAGCAAAGAATGTTGTGA<br>ATAATTGTGAAATAAATTGAAAG
p4MdLOX1<br>*a-2*-M1	GATTACATCTATGTCATCTTAAAGCCAGCTAACTTTCGGGATA<br>AACGATTTTGTAC	GTACAAAATCGTTTATCCCGAAAGTTAGCTGGCTTTAAGATGAC<br>ATAGATGTAATC
p4MdLOX1<br>*a-2*-M2	AATTACGCAGGTGTCATCTTAAAGCCAGCTAACTTTCGGGATA<br>AACGATTTTGTAC	GTACAAAATCGTTTATCCCGAAAGTTAGCTGGCTTTAAGATGAC<br>ACCTGCGTAATT
p4MdLOX1<br>*a-2*-M3	AATTACGCAGGGATTACATCTAAGCCAGCTAACTTTCGGGATA<br>AACGATTTTGTAC	GTACAAAATCGTTTATCCCGAAAGTTAGCTGGCTTAGATGTAAT<br>CCCTGCGTAATT
p4MdLOX1<br>*a-2*-M4	AATTACGCAGGGATTACATCTATGTCATCTTAATTTCGGGATA<br>AACGATTTTGTAC	GTACAAAATCGTTTATCCCGAAATTAAGATGACATAGATGTAA<br>TCCCTGCGTAATT
p4MdLOX1<br>*a-2*-M5	AATTACGCAGGGATTACATCTATGTCATCTTAAAGCCAGCTAA<br>CACGATTTTGTAC	GTACAAAATCGTGTTAGCTGGCTTTAAGATGACATAGATGTAAT<br>CCCTGCGTAATT
p4MdLOX1<br>*a-2*-M6	AATTACGCAGGGATTACATCTATGTCATCTTAAAGCCAGCTAA<br>CTTTCGGGATAA	TTATCCCGAAAGTTAGCTGGCTTTAAGATGACATAGATGTAATC<br>CCTGCGTAATT
Mutant-M1	GATTACATCTATGTCATCTTAAAGCCAGCTAACTTTCGGGATA<br>AATTACCTTATAC	GTATAAGGTAATTTATCCCGAAAGTTAGCTGGCTTTAAGATGA<br>CATAGATGTAATC
